# Single-nucleotide RNA m^6^A mapping in bovine preimplantation development reveals site-specific regulation of RPL12 at zygotic genome activation

**DOI:** 10.1016/j.celrep.2026.117635

**Published:** 2026-07-06

**Authors:** Rajan Iyyappan, Yichi Niu, Yang Li, Hao Ming, Kinga Pajdzik, Noah R. Rakestraw, Piyush K. Jain, Chuan He, Chenghang Zong, Zongliang Jiang

**Affiliations:** 1 Department of Animal Sciences, Institute of Food and Agricultural Sciences, Genetics Institute, University of Florida, Gainesville, FL 32610, USA; 2 Department of Molecular and Human Genetics, Baylor College of Medicine, Houston, TX 77030, USA; 3 Department of Chemistry, Department of Biochemistry and Molecular Biology, and Institute for Biophysical Dynamics, University of Chicago, Chicago, IL 60637, USA; 4 Department of Molecular Genetics and Microbiology, College of Medicine, University of Florida, Gainesville, FL 32610, USA; 5 Department of Chemical Engineering, University of Florida, Gainesville, FL 32610, USA; 6 Health Cancer Center, University of Florida, Gainesville, FL 32610, USA; 7 Howard Hughes Medical Institute, University of Chicago, Chicago, IL 60637, USA; 8 These authors contributed equally; 9 Lead contact

## Abstract

RNA *N*^6^-methyladenosine (m^6^A) is a key regulator of gene expression during early embryogenesis. Using SAC-seq (m^6^A-selective allyl chemical labeling and sequencing), an antibody-independent m^6^A profiling method, we generated the first single-nucleotide-resolution m^6^A map of bovine oocytes and preimplantation embryos. We observed both coordinated and uncoupled relationships between m^6^A modification and expression of protein-coding and noncoding genes. Integrative analysis of the transcriptome, m^6^A epitranscriptome, and translatome revealed dynamic m^6^A remodeling, particularly in ribosomal protein genes. Functional interrogation of a specific m^6^A site within the RPL12 transcript demonstrated that loss of this modification reduces protein synthesis, disrupts translation-related gene expression, impairs zygotic genome activation, and compromises blastocyst formation. Notably, supplementation with wild-type *RPL12* mRNA failed to rescue developmental arrest, suggesting that m^6^A regulates RPL12 function beyond transcript abundance. Overall, these findings provide a valuable single-nucleotide-resolution resource of m^6^A dynamics in mammalian embryogenesis and uncover a site-specific mechanism by which m^6^A regulates translation and developmental competence in early embryos.

## In brief

Iyyappan et al. profile m^6^A at single-nucleotide resolution during bovine preimplantation development and demonstrate that site-specific m^6^A on RPL12 is essential for translation and embryonic progression. This study provides a valuable resource for m^6^A research in bovine embryogenesis and uncovers a critical mechanism by which precise, site-specific m^6^A regulates embryonic development.

## Graphical Abstract

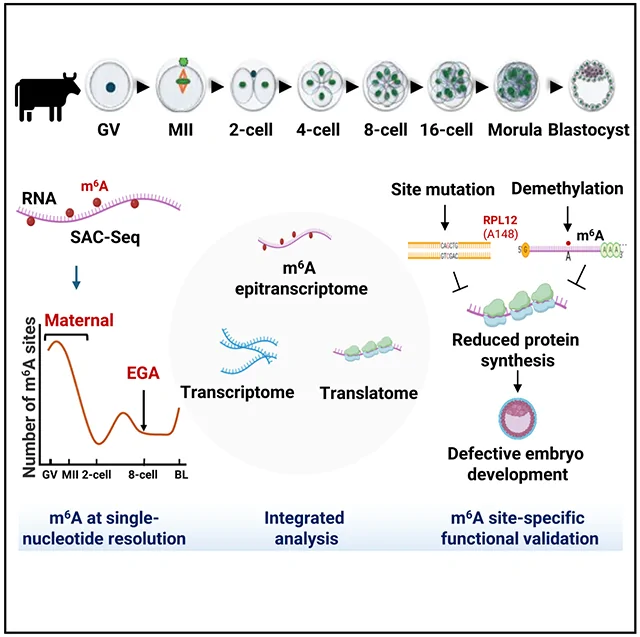

## INTRODUCTION

During preimplantation development, the mammalian embryo undergoes a dramatic maternal-to-zygotic transition (MZT) after fertilization, followed by precise cell fate specification at the blastocyst stage. This process is tightly regulated to ensure the widespread degradation of maternally stored RNAs and proteins, as well as the gradual activation of the embryonic genome.^1,2^ Among key regulatory mechanisms, RNA *N*^6^-methyladenosine (m^6^A) has emerged as a critical modulator of gene expression. As the most abundant internal mRNA modification in eukaryotic mRNA, m^6^A plays essential roles in regulating RNA metabolism, including mRNA stability, splicing, nuclear export, and translation.^3-5^ These effects are mediated by a coordinated network of m^6^A “writers” (METTL3/METTL14), “erasers” (FTO and ALKBH5), and “reader” proteins (YTH domain family proteins), which selectively recognize methylated transcripts and regulate their fate.^5,6^ For example, m^6^A can promote mRNA decay via YTHDF2-mediated recruitment of degradation machinery,^6^ while enhancing translation efficiency through YTHDF1-dependent mechanisms.^7^ In addition, m^6^A influences RNA structure and ribosome dynamics,^8^ thereby regulating gene expression programs in a context-dependent manner. These regulatory functions are particularly important during early embryogenesis, where transcription is limited and post-transcriptional control is essential for developmental progression. m^6^A also plays a major role in early embryonic development by influencing RNA stability and degradation, particularly during the MZT.^4,5,9-11^

To date, transcriptome-wide m^6^A dynamics during mammalian preimplantation development have been characterized in mouse^10,12-14^ and humans,^15^ but not in other mammalian species, including bovine, which has substantial agricultural value and serves as a highly informative large animal model for human early embryonic development.^16-18^ However, profiling m^6^A in early embryos remains technically challenging due to limited material, low sensitivity, and low coverage.

Current embryo m^6^A profiling studies^10,12-15^ have primarily relied on the antibody-based methyl RNA immunoprecipitation sequencing (MeRIP-seq). However, these approaches are limited to identifying m^6^A-enriched regions and are biased toward canonical (DRACH) sequences. Recently, single-nucleotide-resolution m^6^A mapping methods have been developed, including GLORI (glyoxal- and nitrite-mediated deamination of unmethylated adenosines),^19,20^ SAC-seq (m^6^A-selective allyl chemical labeling and sequencing),^21^ eTAM-seq (evolved TadA-assisted N^6^-methyladenosine sequencing),^22^ and CAM-seq (chemical cooperative catalysis-assisted m^6^A sequencing),^23^ providing a quantitative view of m^6^A in biological regulation. Despite these advances, such high-resolution approaches have not yet been applied to mammalian oocytes and embryos. Thus, the functional roles of site-specific m^6^A modifications in regulating key developmental genes during early embryogenesis remain largely unknown.

To overcome this limitation, we applied SAC-seq^21^ to low-input samples and mapped single-nucleotide-resolution m^6^A landscapes across bovine oocytes and preimplantation embryos. Unlike antibody-based approaches such as MeRIP-seq, SAC-seq enables unbiased and motif-independent detection of m^6^A sites, resolving m^6^A dynamics with higher specificity, including transcripts with non-DRACH motifs. Moreover, unlike other single-nucleotide-resolution approaches such as eTAM-seq, GLORI and CAM-seq, which convert all A to I and reduce the 4-letter genome to 3, SAC-seq detects m^6^A as a mutation signature while preserving sequence complexity, which is important for mapping repetitive RNAs.^21^ Our results reveal dynamic, stage-specific patterns of m^6^A deposition across coding and non-coding transcripts, which coincide with major developmental events, including maternal RNA decay and zygotic genome activation (ZGA), as well as changes in translational capacity.

m^6^A is known to regulate mRNA stability, translation, and decay.^3,7,24^ Site- and region-specific m^6^A modifications have been shown to regulate translation efficiency and mRNA stability,^25^ and m^6^A can also influence ribosome dynamics to promote selective transcript decay.^26^ Together, these findings highlight the context-dependent role of m^6^A in gene expression regulation. However, whether m^6^A selectively governs the translation of specific gene classes, particularly ribosomal protein genes (RPGs), during early embryogenesis remains unknown. RPGs, traditionally viewed as structural components of the translational machinery, can also regulate selective translation and developmental competence.^27,28^ Notably, during oocyte maturation and early embryogenesis, when transcription is largely silent, cells rely heavily on translational control to regulate gene expression.^29,30^

To investigate this regulatory layer, we identified a previously uncharacterized m^6^A site in the ribosomal protein L12 (RPL12) transcript that is essential for embryonic development. This finding links m^6^A regulation to ribosome function and global protein synthesis during early development. Together, our results uncover a critical mechanism by which site-specific m^6^A fine-tunes gene expression and translation to support developmental competence, expanding our understanding of RNA modifications in mammalian embryogenesis.

## RESULTS

### Transcriptome-wide mapping of m^6^A in bovine oocytes and preimplantation embryos by SAC-seq

**Figure 1.**
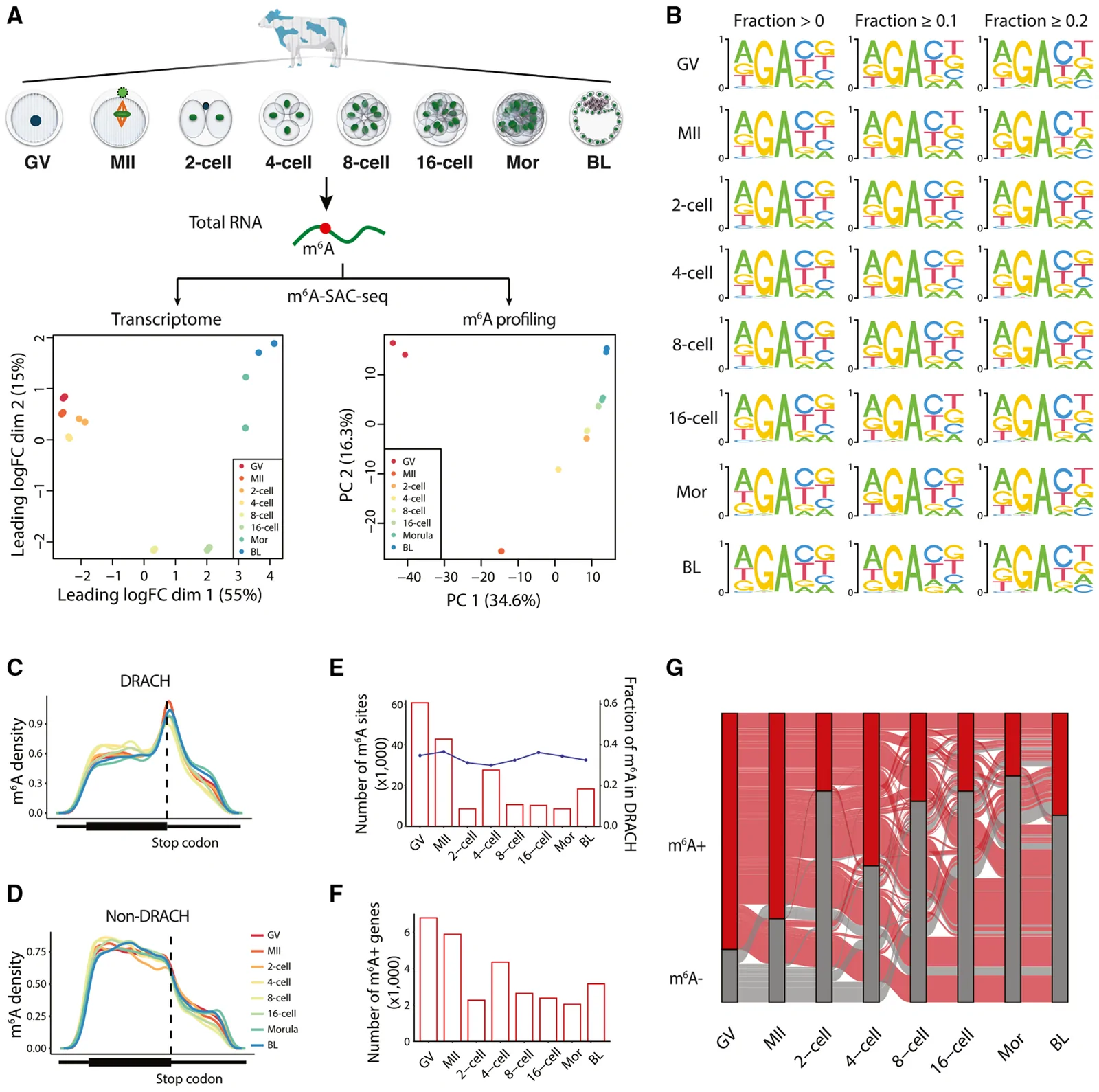
Transcriptome-wide mapping of m^6^A in bovine oocytes and preimplantation embryos by SAC-seq (A) Schematic overview of m^6^A-SAC-seq profiling across bovine oocytes and preimplantation embryo stages, from germinal vesicle (GV) oocyte to blastocyst (BL). Principal-component analysis (PCA) of transcriptome (left) and m^6^A epitranscriptome (right) reveals stage-specific clustering patterns across development. (B) Sequence logos representing nucleotide enrichment around m^6^A sites in different developmental stages. (C and D) Density plots showing the positional distribution of m^6^A sites within transcripts, separated by developmental stages and motif type; DRACH (C) and non-DRACH (D) motifs. Dashed lines indicate stop codon. Black bars below each plot represent transcript regions (5′ UTR, CDS, 3′ UTR). (E) Bar graph showing the number of m^6^A sites detected per stage (red bars) and the fraction of m^6^A sites within canonical DRACH motifs (blue line). (F) Number of m^6^A-modified genes per developmental stage. (G) Sankey diagram showing the transition of transcripts with or without m^6^A modifications (m^6^A^+^ in red, m^6^A^−^ in gray) across developmental stages.

SAC-seq enables single-nucleotide-resolution mapping of m^6^A sites,^21^ overcoming the limitations of antibody-based MeRIP-seq by detecting both canonical (DRACH) and non-canonical motifs without sequence bias. Here, we employed SAC-seq to simultaneously analyze the transcriptome and m^6^A epitranscriptome of bovine oocytes at the germinal vesicle (GV) and meta-phase II (MII) stages, as well as preimplantation embryos at the 2-cell, 4-cell, 8-cell, 16-cell, morula, and blastocyst stages (Figure 1A). For each sample, 200 oocytes or embryos were used, and experiments were performed twice. On average, we obtained approximately 39.3 ± 18.8 million uniquely mapped reads per SAC-seq sample (m^6^A epitranscriptome) and 32.7 ± 9.9 million reads per input control (transcriptome). Principal component analysis (PCA) of both the transcriptome and m^6^A epitranscriptome data showed high consistency between biological replicates and revealed stage-specific patterns of gene expression and m^6^A profiles (Figure 1A).

Motif analysis revealed strong enrichment of the GA motif across developmental stages (Figure 1B), consistent with previously reported SAC-seq performance,^21^ supporting its utility for identifying m^6^A sites in oocytes and early embryos. We next grouped m^6^A sites based on whether they were located within the canonical DRACH motif. On average, 33.5% ± 2.4% of identified m^6^A sites fell within the DRACH motif, ranging from 29.9% at the 4-cell stage to 36.6% at the MII stage (Data S1). Metagene analysis revealed that DRACH-associated m^6^A sites were strongly enriched near stop codons (Figure 1C), consistent with observations in mouse^12^ and humans.^15^ In contrast, m^6^A deposition in non-DRACH motifs was enriched within coding DNA sequences (CDSs) (Figure 1D), highlighting expanded sequence diversity and functional potential beyond canonical motif regions. To validate these observations, independent low-input eTAM-seq analysis confirmed m^6^A sites in both DRACH and non-DRACH sequence contexts for selected transcripts, supporting the authenticity of non-canonical m^6^A sites (Figures S1A and S1B). Motif frequency analysis across different read coverage thresholds showed consistent proportions of DRACH and RRACH motifs, indicating that enrichment of non-DRACH sites is not driven by sequencing depth or calling bias (Figures S1C and S1D).

The total number of identified m^6^A sites across preimplantation stages ranged from 60,678 (GV), 42,825 (MII), 8,598 (2-cell), 27,695 (4-cell), 10,683 (8-cell), 10,263 (16-cell), 8,593 (morula), to 18,320 (blastocyst) (Figure 1E). These sites correspond to 6,779 genes in GV, 5,879 in MII, 2,263 in 2-cell, 4,353 in 4-cell, 2,634 in 8-cell, 2,379 in 16-cell, 2,037 in morula, and 3,156 in blastocyst stages (Figure 1F). Notably, maternal transcripts in oocytes were highly m^6^A-modified compared to later stages (Figures 1E and 1F). These modifications sharply declined after fertilization at the 2-cell stage, indicating a rapid epitranscriptomic switch coincident with MZT. This was followed by two waves of m^6^A re-establishments, one at the 4-cell stage, just prior to bovine major ZGA,^17^ suggesting a potential role in minor ZGA, and another at the blastocyst stage, coinciding with lineage specification. Sankey analysis tracking transcripts with or without m^6^A across preimplantation development showed that most m^6^A-marked transcripts in GV oocytes were retained in MII oocytes, but were largely lost at the 2-cell stage (Figure 1G). A subset of maternal transcripts without m^6^A became methylated at later embryonic stages, particularly in blastocysts (Figure 1G). Overall, these data indicate that maternal transcripts are extensively modified by m^6^A, likely contributing to stability and timely clearance, whereas demethylation of the majority of genes at 8-cells and re-methylation at blastocysts support ZGA and lineage differentiation, respectively. Together, these findings highlight dynamic and tightly regulated m^6^A deposition as a key layer of post-transcriptional control during bovine early embryogenesis.

### m^6^A epitranscriptomic regulation in differentially expressed gene clusters

**Figure 2.**
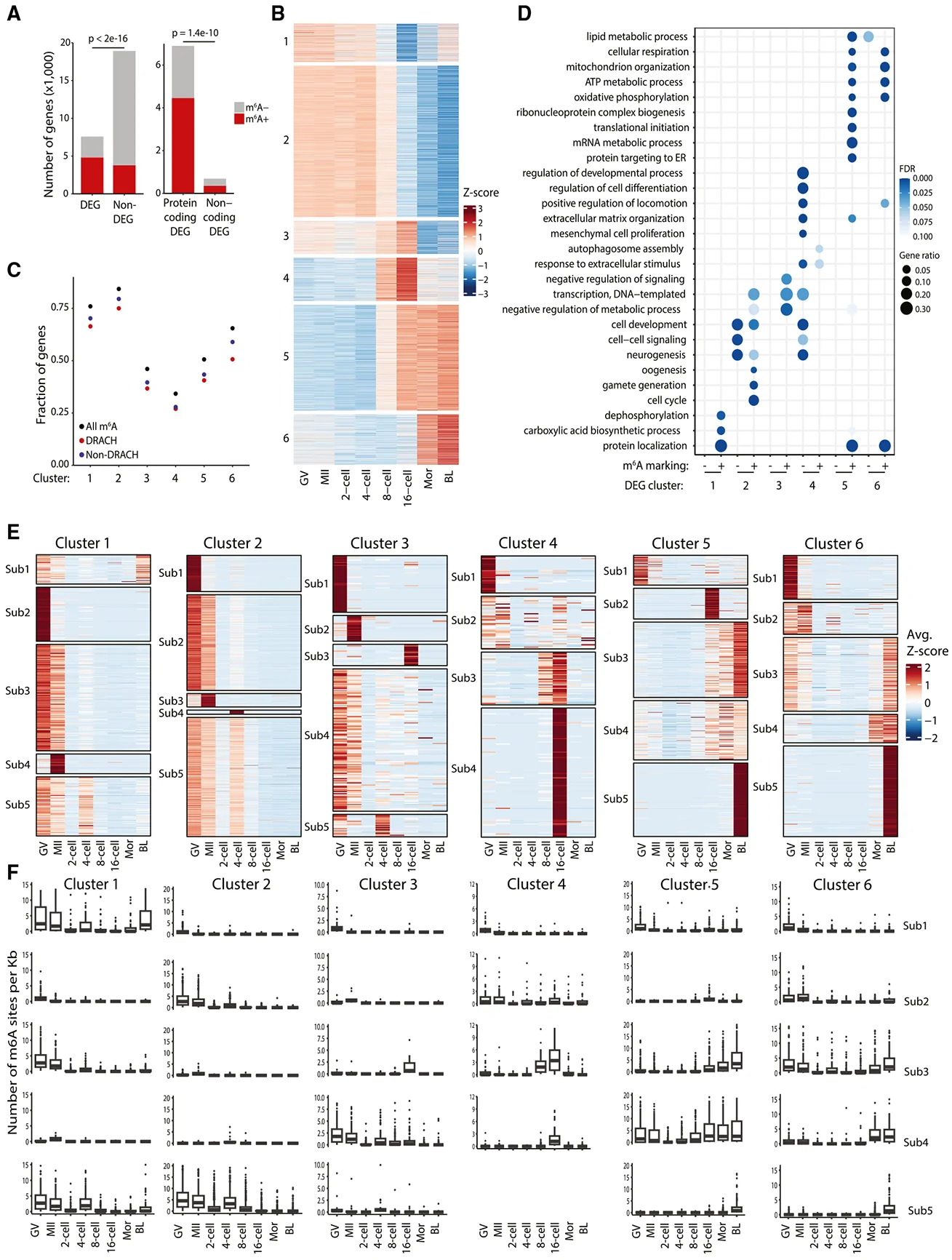
m^6^A epitranscriptomic regulation during bovine preimplantation development (A) Bar plots showing enrichment of m^6^A-modified transcripts among differentially expressed genes and within protein-coding and non-coding DEGs. (B) Heatmap of *Z* score-normalized expression of all DEGs clustered into six groups (1–6) based on temporal expression profiles across oocyte and embryo stages. (C) Fraction of genes within each expression cluster (1–6) carrying m^6^A marks, separated into total m^6^A, DRACH motif-associated, and non-DRACH motif-associated m^6^A sites. (D) Gene Ontology (GO) enrichment analysis of DEGs across expression clusters. DEGs with and without m^6^A marking are compared within each cluster. (E) Heatmaps of *Z* score-normalized m^6^A levels across developmental stages (GV to BL) for five subclusters within each main cluster (1–6). (F) Stage-specific distribution of m^6^A site density (number of m^6^A sites per kb) across subclusters within each cluster. Boxplots show dynamic changes in m^6^A enrichment across developmental stages, highlighting subcluster-specific regulation.

To explore the relationship between m^6^A and gene expression, we analyzed differentially expressed genes (DEGs) across bovine oocytes and preimplantation embryos. A significantly higher proportion of DEGs were m^6^A-modified compared to non-DEGs, with strong enrichment in protein-coding genes (Figure 2A), suggesting preferential marking of dynamically regulated transcripts.

*k*-Means clustering of protein-coding DEGs revealed six distinct expression patterns during preimplantation development (Figure 2B). The fraction of m^6^A-modified genes varied across clusters, with clusters 1, 2, and 6 showing the highest proportions (Figure 2C), indicating functional diversity in stage-specific regulation. Similar proportions were observed for DRACH and non-DRACH sites (Figure 2C). Integration of Figures 2B and 2C revealed that oocyte- and early cleavage-enriched genes are highly m^6^A modified, followed by a sharp decrease at the 8-cell stage and a recovery at the morula and blastocyst stages.

Gene Ontology (GO) analysis of m^6^A-marked DEGs revealed a sequential functional enrichment during developmental progression (Figure 2D). Cluster 1 genes, highly expressed in oocytes, were enriched in dephosphorylation, carboxylic acid biosynthetic process, and protein localization. Cluster 2 genes, active during early cleavage stages, were associated with oogenesis, cell cycle progression, and gamete generation. Clusters 3–6 showed distinct enrichments: cluster 3 in cell-cell signaling and development, cluster 4 in transcriptional regulation and response to extracellular stimuli, cluster 5 in extracellular matrix organization and differentiation, and cluster 6 in translational initiation, ribonucleoprotein complex biogenesis, and mitochondrial metabolism. In contrast, non-m^6^A-marked genes were more frequently associated with signaling and transcriptional regulation pathways (Figure 2D). These differences suggest that m^6^A selectively marks transcripts associated with specific developmental programs.

### Dynamics of m^6^A epitranscriptomic regulation

Transcripts with high m^6^A density are known to be subject to post-transcriptional regulation, including increased decay, altered translation efficiency, and localization changes, depending on the context and interaction with specific m^6^A reader proteins.^6,31,32^ We therefore analyzed m^6^A density dynamics within DEG clusters using subcluster-level analysis (Figures 2E and S2). Distinct temporal patterns of m^6^A enrichment were observed across subclusters (Figure 2F). Some subclusters showed higher m^6^A density during early cleavage stages (Figure 2F, Cluster 1 sub1, 3, and 5, Cluster 2 sub2 and 5, and Cluster 3 sub4), whereas others exhibited increased m^6^A toward morula and blastocyst stages (Figure 2F, Cluster 3 sub3–5 and Cluster 6 sub3–5), indicating stage-dependent regulation. Comparison of m^6^A density and DRACH motif enrichment (Figures 2F and S3A) revealed heterogeneous and stage-dependent relationships rather than a consistent global correlation. In some subclusters, higher m^6^A density was accompanied by lower DRACH motif enrichment; however, this pattern was not uniformly observed. These findings suggest that both canonical and non-canonical targeting mechanisms may contribute to m^6^A deposition in a context-dependent manner. Consistent with this, DRACH-enriched subclusters also showed variable m^6^A density, indicating that motif presence alone does not determine methylation extent.

To control for expression bias, m^6^A density was normalized by transcript length and expression level (sites per kb per reads per kilobase of transcript per million mapped reads (RPKM)) and significant differences across subclusters were retained (Figure S3B). These results indicate that the observed m^6^A dynamics are not driven by transcript abundance. Integration with transcriptomic and translatomic data (Figure S2) further supports coordinated but stage-specific regulation during development.

### Stage-specific m^6^A regulation of non-coding RNAs

**Figure 3.**
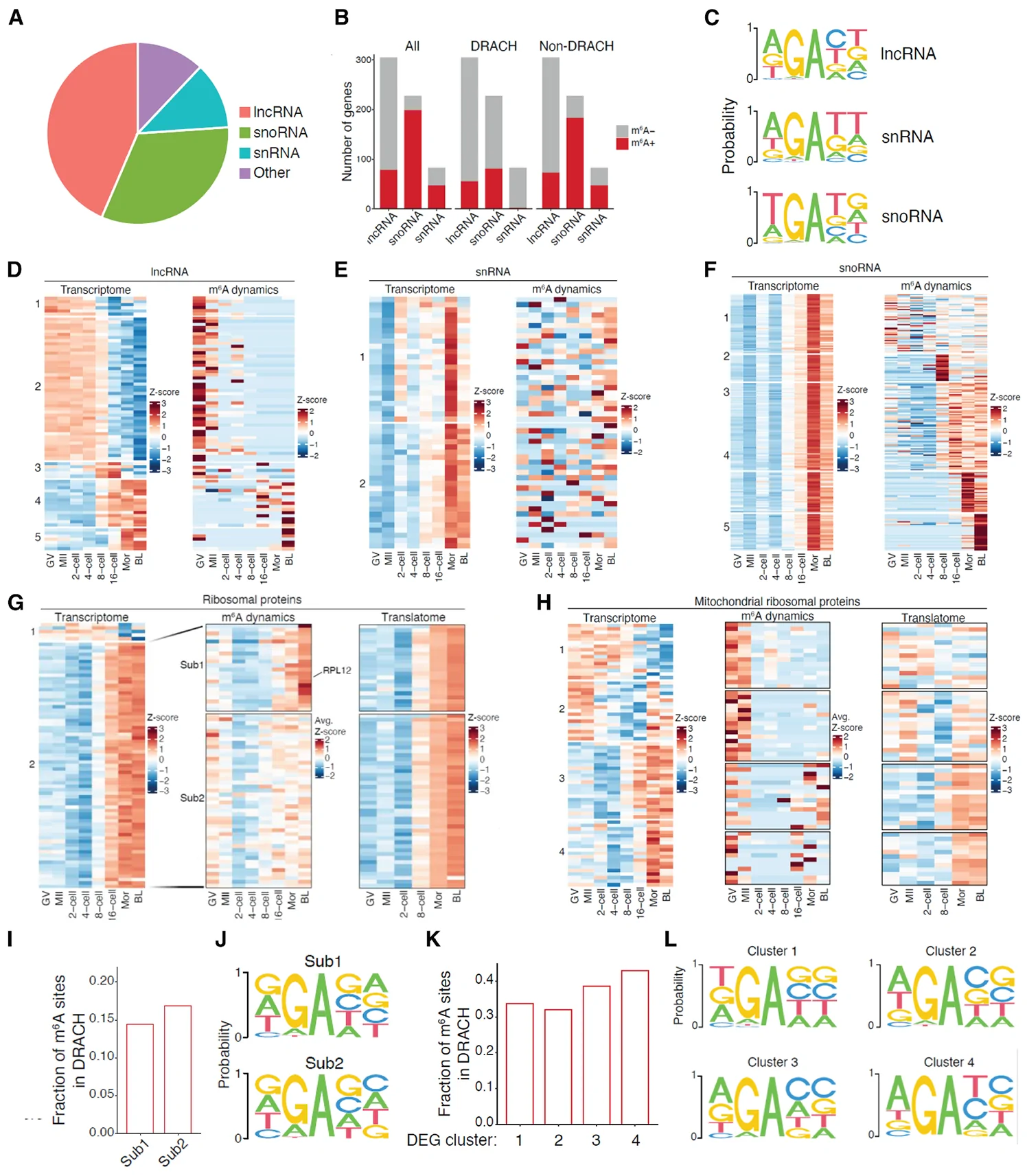
Stage-specific m^6^A regulation of non-coding RNAs and ribosomal protein transcripts (A) The composition of differentially expressed non-coding RNAs. (B) The number of m^6^A+ (red) and m^6^A− (gray) genes across ncRNA classes, separated by all m^6^A, DRACH motif-associated, and non-DRACH motif-associated sites. (C) Sequence logos showing enriched m^6^A motifs in lncRNA, snRNA, and snoRNA classes. (D–F) Heatmaps showing transcriptome expression (left) and m^6^A dynamics (right) across developmental stages; lncRNAs (D) snRNAs (E), and snoRNAs (F). (G) Ribosomal protein genes showing transcriptome expression (left), m^6^A dynamics (middle), and translatome data (right) across embryo stages. Cluster 2 genes are subdivided into Sub1 and Sub2 clusters based on dynamics of m^6^A modification. (H) Mitochondrial ribosomal protein genes showing transcriptome expression (left), m^6^A dynamics (middle), and translatome data (right) across embryo stages. Genes are subdivided into four clusters based on expression trends. (I) The fraction of m^6^A sites in DRACH motifs for Sub1 and Sub2 ribosomal protein clusters. (J) Sequence logos showing enriched m^6^A motifs in Sub1 and Sub2 ribosomal protein clusters. (K) The fraction of m^6^A sites in DRACH motifs for cluster 1-4 mitochondrial ribosomal protein. (L) Sequence logos showing m^6^A motif enrichment for clusters 1–4 of mitochondrial ribosomal protein.

To extend our analysis beyond protein-coding genes, we examined m^6^A dynamics in non-coding RNAs (ncRNAs) (Figure 3A). m^6^A was most prevalent in small nucleolar RNAs (snoRNAs), followed by long non-coding RNAs (lncRNAs) and small nuclear RNAs (snRNAs) (Figure 3B). While DRACH and non-DRACH motifs contributed similarly to lncRNA methylation, non-canonical motifs were significantly enriched in snoRNAs and snRNAs (Figure 3B). Distinct sequence motif enrichments were observed across ncRNA classes, with subtle differences in the flanking nucleotides of the m^6^A sites (Figure 3C).

Analysis of expression and m^6^A abundance across developmental stages revealed distinct patterns for each type of ncRNA (Figures 3D-3F). lncRNAs were highly expressed and m^6^A modified in GV, MII (Clusters 1 and 2), and blastocyst stages (Cluster 5) (Figure 3D), suggesting stage-specific roles. In contrast, snRNAs showed variable m^6^A independent of expression changes (Figure 3E). snRNAs also exhibited asynchronous relationships between expression and m^6^A modification in the majority of subclusters (Figure 3F), with increased m^6^A from 8-cell to blastocyst stages. To further examine whether ncRNA m^6^A dynamics were driven by canonical or non-canonical sites, we stratified lncRNA and snoRNA m^6^A patterns by DRACH and non-DRACH motifs. Both classes contributed to stage-resolved ncRNA dynamics, indicating that the observed changes are not restricted to canonical motif sites (Figures S4A and S4B). We would like to point out that upregulation of snoRNAs reflects their established roles in rRNA modification and ribosome biogenesis, which are critical for expanding protein synthesis capacity in rapidly dividing embryonic cells.^33,34^ Overall, these findings indicate heterogeneous and class-specific regulation of ncRNAs by m^6^A during early embryogenesis.

### Ribosomal protein gene-associated m^6^A regulation

Given the central role of translation control during MZT,^35^ we performed correlative analysis of transcriptome, m^6^A epitranscriptome, and translatome of bovine preimplantation development, focusing on RPGs (GO:0005840). Most RPGs increased expression after the 8-cell stage (Figure 3G). Based on m^6^A dynamics, they were divided into two sub-clusters (sub1 and sub2) (Figure 3G). sub1 showed increased m^6^A after ZGA, whereas sub2 displayed stable m^6^A across oocytes and preimplantation embryos.

Mitochondrial RPGs formed four subclusters (sub1–sub4) (Figure 3H). Sub1 and sub2 showed high expression and m^6^A levels during GV and MII stages, suggesting maternal origin and early marking; however, their transcript and translation levels declined after the 2-cell stage and remained relatively low at later stages. In contrast, sub3 and sub4 showed increased expression and translation from the 8-cell stage onward. Notably, these subclusters did not show prevalent m^6^A modifications, indicating uncoupling between m^6^A and expression or translation efficiency.

We further assessed whether specific m^6^A sequence contexts contribute to differential regulation. Motif analysis revealed a higher fraction of canonical DRACH-associated sites in sub2 compared with sub1 in cytoplasmic RPGs (Figure 3I), and similarly in sub3 and sub4 compared to sub1 and sub2 for mitochondrial RPGs (Figure 3K). This difference suggests that these transcripts may be preferentially targeted by the core m^6^A methyltransferase complex, which typically recognizes motif-dependent sites. Indeed, sequence logo analysis further confirmed these observations, revealing subtle but distinct motif differences between subclusters in both cytoplasmic and mitochondrial ribosomal protein groups (Figures 3J and 3L). Notably, sub2 motifs closely matched the canonical DRACH consensus, but sub1 motifs displayed a different motif pattern, particularly at the +1 and +2 positions flanking the methylated adenosine (Figure 3J). These findings suggest that functionally or temporally distinct subsets of ribosomal transcripts may be associated with different m^6^A writer or reader complexes, adding an additional layer of epitranscriptomic specificity during early embryonic development.

Together, these results indicate that m^6^A dynamics in RPGs may contribute to post-transcriptional regulation in a context-dependent manner, rather than directly determining transcript or translation levels. To further investigate functional relevance, we selected RPL12 for mechanistic study.

### Site-specific m^6^A modification of RPL12 is required for ZGA and blastocyst formation

**Figure 4.**
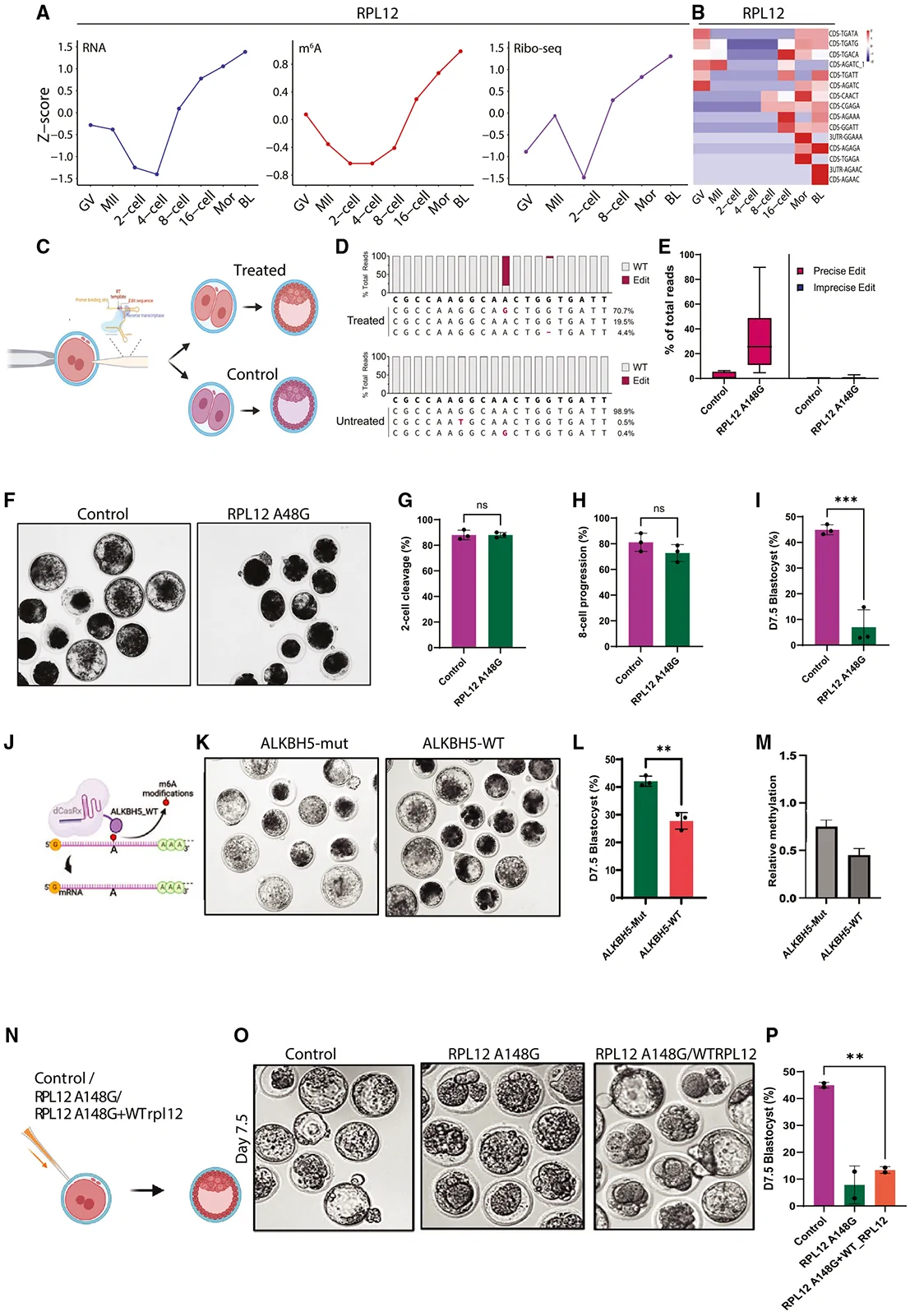
Site-specific m^6^A modification of RPL12 is required for ZGA and blastocyst formation (A) Trends of RPL12 transcript expression (left), m^6^A modification level (middle), and ribosome occupancy (Ribo-seq) (right) across developmental stages. (B) The stage- and site-specific m^6^A modification of RPL12 transcripts. (C) Schematic of editing strategy: PEMax fusion is delivered into zygotes to edit the m^6^A site on RPL12 (148^th^ A to G). (D) Representative amplicon sequencing confirms efficient m^6^A site editing in RPL12 at 148^th^ position A>G. (E) Boxplots showing the percentage of total reads containing precise and imprecise edits at the RPL12 A146G site in control versus edited embryos. (F) Representative bright-field images showing developmental differences between control and edited embryos. (G–I) Quantitative data showing the 2-cell, 8-cell, and blastocyst rate of control and RPL12-edited embryos. Data are presented as the mean ± SEM (*n* = 3). Statistical significance was determined using an unpaired t test. *** *p* < 0.001. (J) Schematic of demethylating strategy: dCas13-ALKBH5 fusion is delivered into zygotes to demethylate the m^6^A site on RPL12. (K) Representative bright-field images showing developmental differences between control (ALKBH5-mut-catalytically inactive) and edited (ALKBH5-WT) embryos. (L) Quantitative data showing the blastocyst rate of control and RPL12-edited embryos. Data are presented as the mean ± SEM (*n* = 3). Statistical significance was determined using an unpaired t test. ** *p* < 0.01. (M) Bar graph showing reduced enrichment of m^6^A at the targeted site in RPL12 following ALKBH5-based demethylation compared to control embryos, as measured by quantitative reverse-transcription PCR. (N) Schematic showing rescue strategy: zygotes were injected with wild-type RPL12 mRNA alongside editing reagents to restore normal RPL12 expression in edited embryos. (O) Representative bright-field images show the blastocyst morphology in the rescue group compared to base-edited embryos. (P) Quantification of blastocyst development rate shows partial or no rescue in RPL12 WT-injected embryos relative to unrescued edited embryos. Data are presented as the mean ± SEM (*n* = 2). Statistical significance was determined using an unpaired t test. ** *p* < 0.01.

As global m^6^A profiling revealed distinct regulatory patterns among RPGs during preimplantation development (Figures 3G-3L), we next investigated whether a single site-specific m^6^A could have functional consequences for embryonic development. We selected *RPL12* for detailed analysis (Figure 4A), as it contains a highly conserved, high-confidence m^6^A site at the 148^th^ adenosine (148^th^ A) within the coding sequence, with increased methylation from 8-cell to blastocyst stages (Figure 4B), coinciding with major ZGA and lineage specification. RPL12 encodes a component of the 60S ribosomal subunit involved in protein synthesis, and previous studies have linked ribosome function to embryonic development.^36-38^ However, its role in ZGA, and specifically the function of this m^6^A site (m^6^A-RPL12), has not been previously characterized.

To investigate this, we used CRISPR-based prime editing (PEMax)^39-41^ to precisely mutate the methylated adenosine to guanosine (A148G) in zygotes (see STAR Methods), thereby preventing m^6^A deposition at this site (Figure 4C). Amplicon sequencing confirmed efficient and precise editing with minimal off-target effects (Figures 4D and 4E). Embryos carrying the A148G mutation developed normally to the 2- to 8-cell stage but exhibited impaired development beyond 8-cell stage and a marked reduction in blastocyst formation compared with controls (Figures 4F-4I). These results indicate that loss of this single m^6^A site compromises ZGA and blastocyst formation.

To confirm that the observed phenotype was specifically due to loss of m^6^A rather than the nucleotide substitution itself, we performed site-specific m^6^A demethylation using dCas13Rx-ALKBH5 (m^6^A demethylase) delivered into zygotes (see STAR Methods) to selectively erase m^6^A at the same 148^th^ A site in *RPL12* mRNA (Figure 4J). This demethylation (ALKBH5-WT) treatment also impaired embryonic development, although the phenotype was less severe than that observed with A148G mutation, likely due to incomplete demethylation efficiency. In contrast, embryos injected with the catalytically inactive mutant (ALKBH5-mut) developed normally (Figures 4K and 4L). Site-specific m^6^A-qPCR confirmed a significant reduction in methylation at the target site following ALKBH5 treatment (Figure 4M), which was further supported by eTAM-seq analysis showing consistent partial demethylation (Figure S5).

To determine whether restoring *RPL12* expression could rescue the developmental defect, we co-injected zygotes with the PEMax system targeting A148G and exogenous wild-type RPL12 mRNA lacking m^6^A (Figure 4N, see STAR Methods). Despite increased total RPL12 transcript levels, supplementation of unmodified mRNA did not rescue blastocyst formation or developmental progression compared with the edited controls (Figures 4O and 4P), indicating that RPL12 transcript abundance alone is insufficient and that the m^6^A modification itself is required for functional rescue.

Together, these results demonstrate that m^6^A modification of RPL12 is essential for normal embryonic development, and that disruption of this site either by base substitution or by site-specific demethylation impairs developmental progression.

### Loss of m^6^A on RPL12 impairs protein synthesis in early embryos

**Figure 5.**
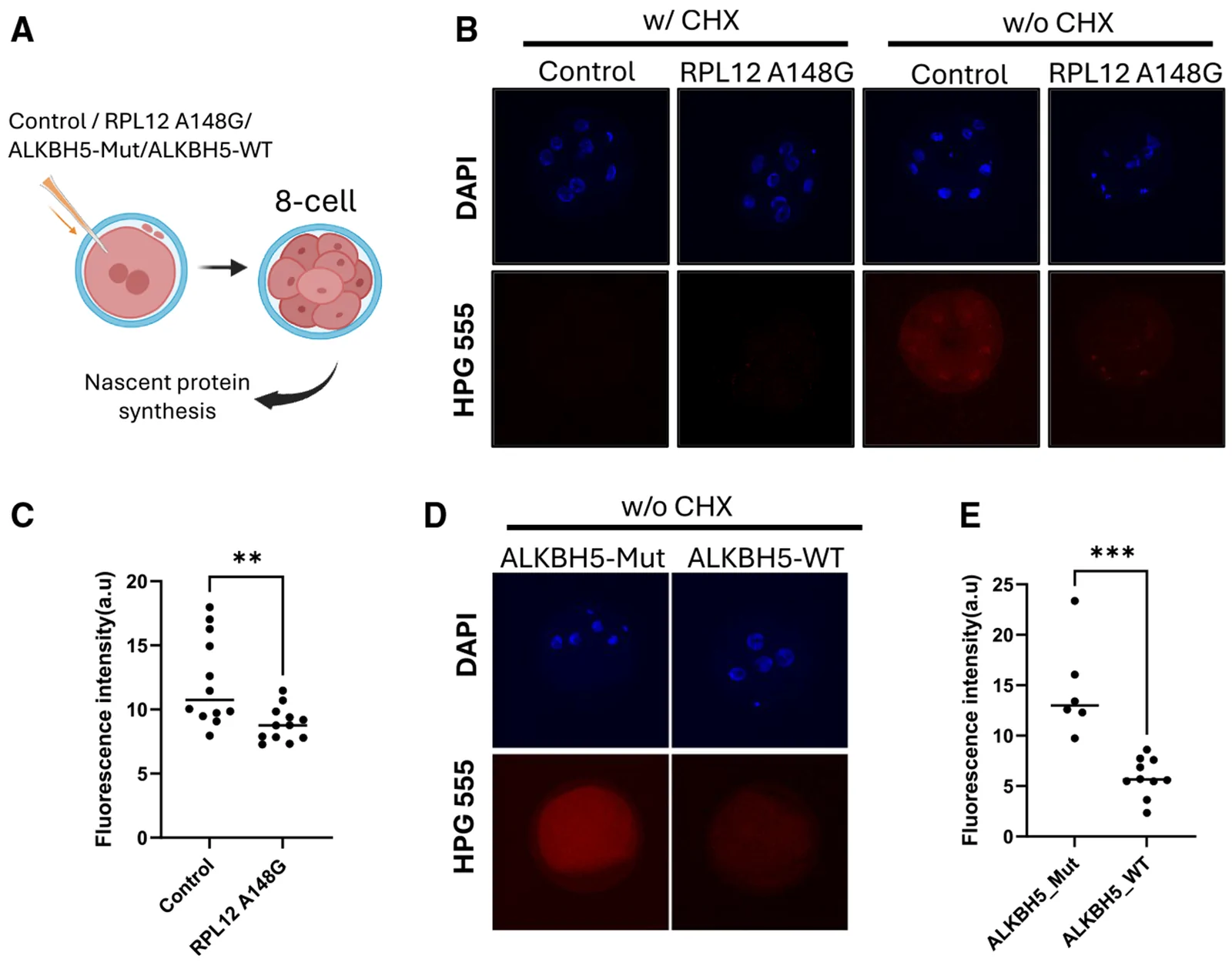
Loss of m^6^A on RPL12 impairs protein synthesis in early embryos (A) Schematic and experimental design for total protein synthesis assay using Click-iT HMG incorporating in 8-cell stage embryos following RPL12 editing. (B) Representative immunofluorescence images showing HMG incorporation (red) and nuclear staining (blue) in control and RPL12 A148G-edited groups. Cycloheximide-treated embryos were used as a negative control. (C) Quantification of HMG signal intensity shows reduced global translation in RPL12-A148G-edited embryos. Data are presented as the mean ± SEM. Statistical significance was determined using a two-tailed Mann–Whitney test. ** indicates *p* < 0.01. (D) Representative immunofluorescence images showing HMG incorporation (red) and nuclear staining (blue) in control and RPL12 demethylated groups. (E) Quantification of HMG signal intensity shows reduced global translation in RPL12 demethylated embryos. Data are presented as the mean ± SEM. Statistical significance was determined using a two-tailed Mann–Whitney test. *** indicates *p* < 0.001.

To investigate the mechanism underlying the role of m^6^A-RPL12 in preimplantation development, we first assessed whether loss of this modification affects global protein synthesis, given the function of RPL12 in translation. We performed a global translation assay using the Click-iT L-homopropargylglycine (HPG) labeling (see STAR Methods) at the 8-cell stage (Figure 5A). Embryos carrying the A148G mutation showed markedly reduced HPG incorporation, indicating a strong decrease in nascent protein synthesis. Signal intensity was comparable to that observed in cycloheximide-treated embryos, which serve as a negative control for active translation (Figure 5B). Quantification confirmed a significant reduction in global translation following loss of m^6^A at RPL12 (Figure 5C).

To validate this result using an independent approach, we examined embryos subjected to dCas13Rx-ALKBH5-mediated demethylation. Consistently, ALKBH5-WT-treated embryos exhibited reduced global protein synthesis, whereas embryos treated with catalytically inactive ALKBH5-mut showed normal translation levels (Figures 5D and 5E).

Together, these findings demonstrate that loss of m^6^A at RPL12 impairs protein synthesis, underscoring the critical role of site-specific m^6^A in regulating ribosomal protein function during early embryogenesis.

### m^6^A-RPL12 regulates post-transcriptional stability and translation of ribosomal transcripts

**Figure 6.**
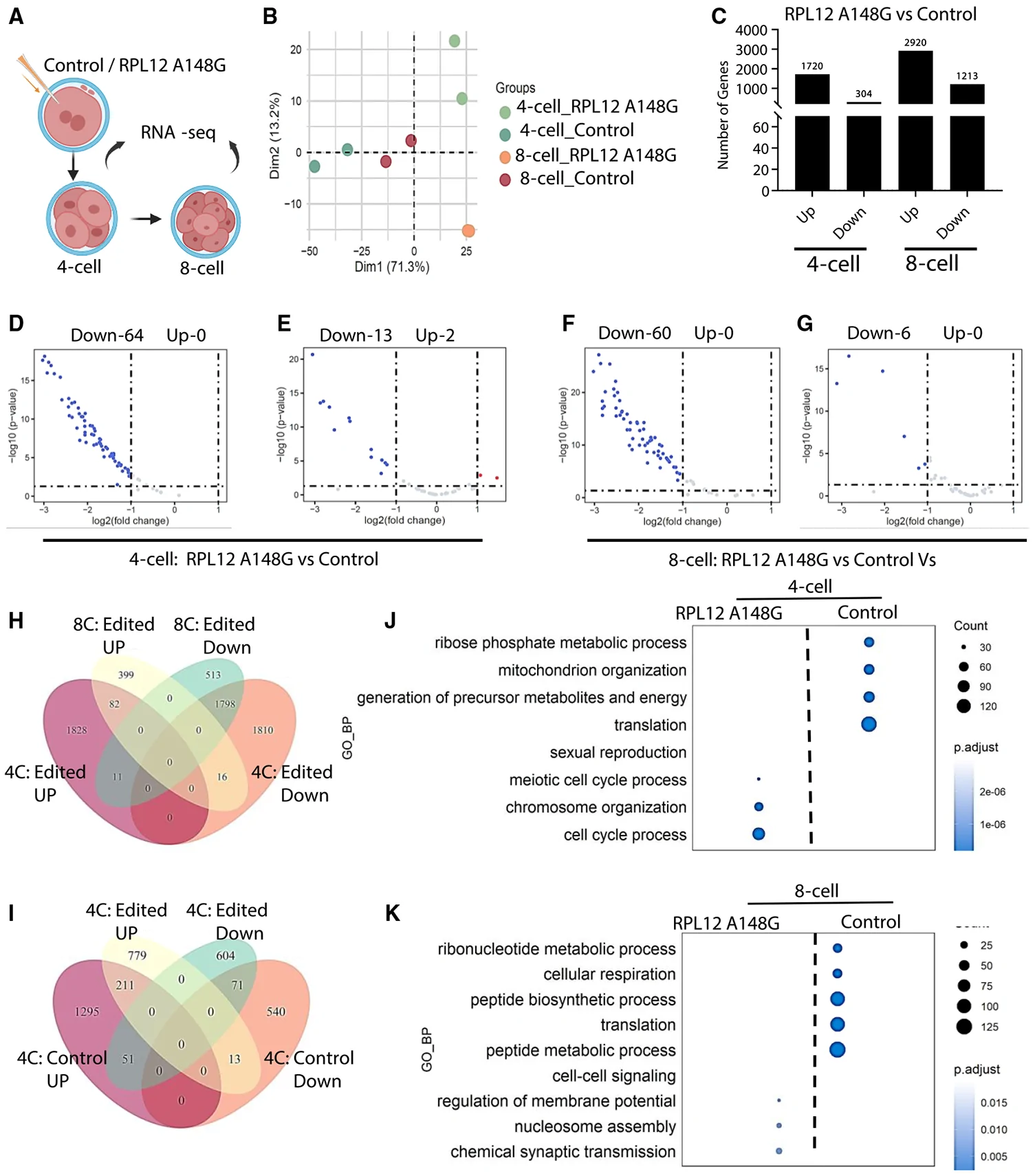
m^6^A-RPL12 regulates post-transcriptional stability and translation of ribosomal transcripts (A) Schematic representation of the experimental design: control and RPL12 A148G-edited bovine embryos were collected at the 4- and 8-cell stages for total RNA-seq. (B) PCA of transcriptomes from control and edited embryos at both stages. (C) Differentially expressed total transcripts (refer Figure S6) in 4- and 8-cell stages. (D and E) Volcano plots comparing ribosomal protein genes (D) and translation initiation/elongation factors (E) between RPL12 A148G-edited and control embryos at the 4-cell stage. (F and G) Volcano plots comparing ribosomal protein genes (F) and translation initiation/elongation factors (G) between RPL12 A148G-edited and control embryos at the 8-cell stage. (H and I) Venn diagrams showing stage-specific overlap of differentially expressed genes in RPL12-edited embryos and control across the 4- and 8-cell stages. (J and K) GO enrichment analysis for differentially expressed genes in control vs. edited embryos at the 4- (J) and 8-cell (K) stages.

To examine the broader impact of m^6^A-RPL12 disruption on embryonic gene expression, we performed RNA sequencing (RNA-seq) analysis on control and PEMax-edited embryos at the 4- and 8-cell stages (Figure 6A, Data S1). PCA revealed clear separation between control and edited groups, as well as between developmental stages, indicating a global transcriptomic shift associated with loss of m^6^A-RPL12 (Figure 6B). Notably, m^6^A-RPL12 disruption reduced the number of DEGs between the 4- and 8-cell transition (Figure 6C), suggesting impaired activation of ZGA-associated transcriptional programs.

Given the observed reduction in protein synthesis (Figures 5B and S6A-S6D), we focused on genes involved in translation, including ribosomal proteins (GO:0005840) and translation initiation and elongation factors (GO:0006412). In control embryos, only a small number of RPGs (*n* = 5) (Figure S6E) and translational factors (*n* = 4) (Figure S6F) were modestly upregulated between the 4- and 8-cell stages, consistent with the onset of translational activation during this window.^35^ In contrast, m^6^A-RPL12-edited embryos showed aberrant upregulation of 22 RPGs at the 4-cell stage, followed by a failure to further increase expression at the 8-cell stage (Figure S6G), indicating disruption of normal temporal regulation. Translation initiation and elongation factors remained relatively stable (Figure S6H).

Direct comparison between control and edited embryos revealed that at the 4-cell stage, 64 RPGs and 13 translation-related factors were significantly downregulated in edited embryos (Figures 6D and 6E). This suppression persisted at the 8-cell stage, with 60 RPGs and 6 translation factors remaining significantly reduced (Figures 6F and 6G). Venn diagram analysis revealed minimal overlap in DEGs between stages, supporting the stage-specific effects of m^6^A-RPL12 disruption (Figures 6H and 6I). GO analysis further showed that mitochondrial organization, metabolic precursor generation, and translation were among the most affected pathways at both stages (Figures 6J and 6K). Pathways related to meiosis, chromosome organization, and cell cycle progression were also disrupted, suggesting broader developmental consequences beyond translation alone.

Together, these data demonstrate that m^6^A-RPL12 is required for proper activation of translation-related transcripts and maintenance of translational capacity during ZGA, thereby ensuring normal embryonic development.

## DISCUSSION

Despite recent progress in understanding transcriptome-wide m^6^A in mammalian preimplantation embryos,^10,12-15^ the landscape of m^6^A dynamics at single-nucleotide resolution during this critical period remains incomplete. Such resolution is necessary to characterize the functions of site-specific m^6^A marks, particularly those exhibiting pronounced dynamics during MZT. In this study, we employed SAC-seq to simultaneously characterize the m^6^A epitranscriptome and transcriptome from the same samples, generating the first single-base-resolution m^6^A landscapes across bovine oocytes and preimplantation embryos.

Through comprehensive bioinformatic analysis, several key insights emerged. First, m^6^A modification was widespread and highly dynamic during preimplantation development, with elevated m^6^A levels in GV and MII oocytes, followed by a sharp decrease immediately after fertilization, coinciding with MZT.^17,35^ This pattern is consistent with observations in mouse and human embryos,^12,15^ where m^6^A has been implicated in maternal mRNA clearance and zygotic transcript stabilization.^4,9^ Second, m^6^A was enriched among DEGs, particularly those involved in translation, mitochondrial metabolism, and cell cycle regulation, suggesting that m^6^A acts as a selective regulator of developmental transcript fate. Within DEG clusters, subcluster-based analysis further revealed distinct dynamic patterns of m^6^A modification associated with stage-specific biological functions. Notably, transcripts activated after ZGA were enriched in translation-related processes, which are essential for developmental competence.^42^ We further observed that transcripts with higher m^6^A site density often showed increased expression at developmental stages where their functions become prominent, suggesting that m^6^A density may fine-tune translational efficiency and/or RNA stability in a context-dependent manner. However, in many subclusters, changes in m^6^A density were not synchronized with gene expression changes, indicating partial uncoupling between epitranscriptomic and transcriptional regulation. Third, we characterized m^6^A in non-coding transcripts and observed dynamic, class-specific enrichment patterns in lncRNAs, snRNAs, and snoRNAs. Finally, to further explore m^6^A regulation of ribosome-associated gene sets, we integrated transcriptome, m^6^A epitranscriptome, and translatome data.^35^ These analyses showed that m^6^A contributes to the regulation of ribosome-related gene sets during early embryogenesis, potentially influencing translational dynamics in a stage- or context-specific manner, thereby supporting models of ribosome heterogeneity.^28,36^

Interestingly, we observed limited concordance between m^6^A dynamics and gene expression changes across developmental stages, differing from trends reported in mouse and human systems where m^6^A often correlates with transcript abundance. Several factors may explain this discrepancy. First, m^6^A regulates multiple layers of RNA metabolism, including translation efficiency and mRNA stability, which may not directly translate into steady-state transcript changes. Second, early embryogenesis is dominated by post-transcriptional regulation, particularly during MZT, where translational control may predominate over transcriptional output. Third, species-specific differences between bovine and other mammals may contribute to distinct m^6^A-expression relationships. Finally, methodological differences, including SAC-seq single-nucleotide resolution versus peak-based approaches, may also affect correlation patterns. Together, these findings suggest that m^6^A-mediated regulation during early embryogenesis extends beyond transcriptional control and involves complex, stage-specific post-transcriptional mechanisms.

To demonstrate gene- and site-specific functional effects, we selected RPL12, an RPG harboring a dynamically regulated m^6^A site essential for developmental progression. Both prime editing and dCas13Rx-ALKBH5-mediated demethylation at the A148 m^6^A site of *RPL12* led to impaired ZGA and reduced blastocyst formation, demonstrating that loss of m^6^A at this single site is sufficient to compromise embryo development. Remarkably, supplementation with wild-type *RPL12* mRNA failed to rescue this phenotype, suggesting that m^6^A regulates developmental competence not through transcript abundance, but likely through modulation of translation efficiency or ribosomal-binding protein recruitment, consistent with prior studies.^7,43^ Consistently, global translation assays showed that RPL12 m^6^A disruption or demethylation reduced protein synthesis, aligning with transcriptomic analyses demonstrating coordinated downregulation of ribosomal genes, translation factors, and metabolic regulators in edited embryos. This transcriptional dysregulation was evident as early as the 4-cell stage and persisted through the 8-cell stage, reinforcing the role of m^6^A as a temporal regulator of gene expression and protein synthesis during early embryogenesis. These findings support emerging models in which m^6^A regulates not only RNA stability and translation efficiency but also overall translational output during key developmental transitions.^7,12,44,45^ While HPG incorporation and transcriptomic analyses strongly suggest that m^6^A at RPL12 modulates global translation, we were unable to directly measure RPL12 protein levels or ribosome association due to limited material and the lack of suitable bovine-specific antibodies. Future studies using stage-specific RPL12 quantification and low-input polysome profiling will be essential to fully elucidate the mechanism by which this m^6^A site regulates translational output.

Importantly, this study provides functional evidence that a single m^6^A site can influence broader transcriptomic programs and contributes to early developmental failure. Early embryonic loss is a major cause of infertility in both humans and cattle. Although *in vitro* fertilization (IVF) is widely used to treat infertility, more than 50% of IVF embryos fail to progress to the blastocyst stage in both humans and cattle. Moreover, the developmental competence of IVF embryos after transfer remains lower than that of *in vivo*-derived embryos in cattle.^46^ Environmental stressors such as *in vitro* culture conditions can induce aberrant molecular changes in embryos, leading to developmental arrest.^47^ It is therefore possible that such stress also induces abnormal m^6^A patterns in IVF embryos during this highly plastic preimplantation period. Our findings suggest that identifying and functionally validating beneficial m^6^A sites may improve embryo competence and that targeted modulation of m^6^A in IVF embryos could represent a potential strategy to enhance fertility outcomes.

Overall, our study reveals a critical role for m^6^A RNA methylation in orchestrating molecular transitions during bovine preimplantation development. This work provides an unprecedented resource for exploring m^6^A regulation in oocyte maturation and preimplantation development.

### Limitations of the study

Our experimental approach has several limitations. First, due to the limited availability of oocytes and preimplantation embryos, both RNA-seq and SAC-seq experiments were performed in duplicate biological samples. Although the datasets are of high quality and internally consistent, additional biological replicates could further strengthen robustness. Second, embryos were generated by IVF, which may introduce stress-associated epitranscriptomic changes compared with *in vivo* development. In addition, SAC-seq was performed on pooled embryos, which may obscure cell-to-cell and lineage-specific variability. Third, eTAM-seq validation showed differences in relative enrichment patterns for some candidate m^6^A sites compared with SAC-seq. However, both SAC-seq and eTAM-seq are relatively recent technologies, and neither provides 100% accuracy, bias-free quantification of m^6^A. Fourth, although this study provides a comprehensive m^6^A landscape in bovine preimplantation development, functional validation was primarily focused on RPL12. Future studies should investigate additional m^6^A-modified genes, including both RPL and RPS ribosomal subunit genes, and determine how perturbations of these regulatory layers contribute to early embryonic loss. Finally, the heterogeneous and dynamic patterns observed highlight that both the regulation and functional consequences of m^6^A are highly context-dependent and complex. Large-scale gene- and site-specific functional investigations will be required in future studies.

## STAR★METHODS

### EXPERIMENTAL MODEL AND STUDY PARTICIPANT DETAILS

No live animals were involved in this study. Bovine oocytes were produced from ovaries collected from slaughterhouse-derived bovine reproductive tracts and different stages of embryos were produced by *in vitro* fertilization. Because embryos cannot be sexed prior to experimental treatments, the analysis of pooled embryos at different developmental stages has ensured a representation of both male and female embryos.

### METHOD DETAILS

#### Bovine oocytes and *in vitro* embryo production

Bovine ovaries were collected from slaughterhouse-derived bovine reproductive tracts, and cumulus-oocyte complexes (COCs) were aspirated from 3 to 5 mm follicles using an 18-gauge needle. Germinal vesicle stage oocytes (GV oocytes) were collected as cumulus-oocyte complexes (COCs) aspirated from slaughterhouse ovaries. Only COCs with a homogeneous cytoplasm and multiple cumulus cell layers were selected for *in vitro* maturation (IVM). Maturation was performed in BO-IVM medium (IVF Bioscience) at 38.5°C with 6% CO_2_ for 22–23 h. After maturation, cumulus cells were removed, and nuclear maturation was confirmed by assessing the presence of the first polar body under a light microscope. For *in vitro* fertilization (IVF), cryopreserved semen from Holstein bulls with proven fertility was processed using BO-SemenPrep medium (IVF Bioscience), and the final sperm concentration was adjusted to 2 × 10^6^ spermatozoa/mL. Matured oocytes were co-incubated with sperm in fertilization medium at 38.5°C in 6% CO_2_ for 18 h. Following fertilization, presumptive zygotes were washed and cultured in synthetic oviduct fluid, BO-IVC medium (IVF Bioscience), in groups of approximately 30 embryos per 50-μL droplet overlaid with mineral oil. Embryos were incubated at 38.5°C in a benchtop incubator (WTA, Cravinhos, SP, Brazil) under a humidified atmosphere of 5% CO_2_, 5% O_2_, and 90% N_2_ until day 7.5 post-fertilization. Cleavage rates were assessed on day 2, and blastocyst formation was evaluated on day 7.5 to determine developmental competence. This optimized embryo production system was used for downstream epitranscriptomic analysis, including m^6^A profiling using SAC-seq.

#### RNA isolation and SAC-seq

Total RNA was isolated from bovine oocytes and early embryos at different developmental stages using TRIsolution (Sigma-Aldrich) according to the manufacturer’s protocol, with modifications to optimize RNA recovery from small sample sizes. Approximately 200 oocytes or embryos were pooled per replicate for RNA extraction. Briefly, samples were lysed in TRIsolution, followed by phase separation with chloroform. The aqueous phase was collected, and RNA was precipitated with isopropanol, washed with ethanol, and resuspended in nuclease-free water. RNA purity and concentration were assessed using a NanoDrop spectrophotometer (Thermo Fisher Scientific), and integrity was evaluated using an Agilent Bioanalyzer 2100 (Agilent Technologies). Only samples with RNA integrity numbers (RIN) above 7.0 were used for downstream analysis. Due to the low RNA input and the unavailability of a bovine-specific ribosomal RNA depletion kit, we proceeded without an rRNA depletion step. Instead, total RNA was directly subjected to SAC-seq (Selectively Allyl-Chemically Labeled m^6^A Sequencing) to achieve single-nucleotide resolution detection of m^6^A modifications. The RNA underwent selective allyl-chemical labeling of m^6^A sites, followed by reverse transcription using a modified primer designed to introduce mutation signatures indicative of m^6^A presence. cDNA libraries were prepared using the NEBNext Ultra II RNA Library Prep Kit (New England Biolabs) and sequenced on an Illumina NovaSeq 6000 platform to generate high-depth paired-end reads. Bioinformatic analysis was conducted using a custom computational pipeline to identify m^6^A sites and assess their dynamic regulation during early bovine embryonic development. The SAC-seq data were analyzed in the context of maternal RNA clearance and zygotic genome activation, providing novel insights into the epitranscriptomic mechanisms regulating bovine embryogenesis.

#### SAC-seq data analysis

##### Data preprocessing:

Raw sequencing data were trimmed using Cutadapt (v5.0) as described previously.^48^ Briefly, the 6-bp UMI sequence and following 5-bp RT tail were firstly trimmed from Read 2 and appended to the read name. The adapter sequences were trimmed from both ends of Read 1 and Read 2. Low quality bases were trimmed with parameter “-q 20”. Reads with a final length <15 bp were discarded.

##### Spike-in analysis and calibration curve for m^6^A stoichiometry estimation:

Trimmed reads were mapped to spike-in RNA using Bowtie2^49^ (v2.2.8) and converted to FASTA format using Samtools^50^ (v1.18). The mapped reads were next assigned to each spike-in RNA sequence using blastn^51^ (v2.3.0+). The conversion rate of m^6^A/A base for each spike-in RNA was then calculated with a custom Python script. Calibration curves were then fit using stats.linregress function from Scipy package. The motifs with a negative slope were excluded from the following analyses.

##### Read mapping, mutation calling and identification of m^6^A sites:

Trimmed reads were mapped to bosTau9 (ARS_UCD1.3) using STAR^52^ (v2.7.9a). After removing the unmapped reads, PCR duplicates were identified and discarded based on UMI sequence and mapping coordinates by using UMI-Tools^53^ (v1.1.6). Variant calling was then performed by Samtools mpileup. All called variants were then filtered by the following criteria: 1) the corresponding loci covered by ≥ 10 reads in both treated and input libraries, 2) variants supported by ≥ 3 reads in each treated library, 3) average mutation ratio in input libraries is <0.01, 4) average mutation ratio in treated libraries is ≥0.05. The identified m^6^A sites were annotated by using variant-effect package (https://github.com/y9c/variant). To calculate the modification fraction, we then normalized the mutation ratio of each m^6^A site using the fitted calibration curve of the corresponding motif. The metagene profiles of detected m^6^A sites were generated using metaPlotR package.^54^

##### RNA-seq data analysis:

Raw sequencing reads were first trimmed as described above. We next mapped the trimmed reads to bosTau9 (ARS_UCD1.3) using STAR (v2.7.9a). Uniquely mapped reads were then assigned to genes using HTseq-count^55^ (v2.0.5) with parameters “-s no -t exon -m intersection-strict”. Normalization and differential gene expression analyses between adjacent time points were performed using edgeR package with a threshold of log_2_ fold change >1 and False discovery rate (FDR) < 0.05. Multiple testing correction was performed using the Benjamini–Hochberg method.^56^ Differentially expressed genes (DEGs) were clustered according to their expression dynamics across different time points using k-means clustering algorithm. To identify the sub-clusters of DEGs based on m^6^A modification, we first scaled the modification fractions of each m^6^A site across different time points. Next, we computed the average m^6^A modification fraction for each gene at different time points by aggregating the scaled modification fractions of all associated m^6^A sites. m^6^A marked DEGs were then clustered into sub-clusters based on their m^6^A modification dynamics across different time points using k-means clustering algorithm.

#### eTAM-seq analysis

RNA from oocytes and embryos were processed using a targeted amplicon eTAM-seq workflow. Cells were lysed in TRIzol reagent (Thermo Fisher Scientific, Cat# 15596026), and total RNA was purified using the Direct-zol RNA MiniPrep kit (Zymo Research, Cat# R2052) with on-column DNase I digestion provided with the kit. Purified RNA was subjected to a one-pot fragmentation and deamination reaction in deamination buffer (50 mM Tris-HCl pH 7.5, 25 mM KCl, 2.5 mM MgCl_2_, 2 mM DTT, and 10% glycerol) containing SUPERase·In RNase inhibitor (Thermo Fisher Scientific, Cat# AM2694). RNA was fragmented by heating at 94°C for 3 min followed by immediate cooling on ice. TadA deaminase was then added, and reactions were incubated at 53°C for 1 h followed by two additional incubations at 44°C for 1 h each. RNA was purified using the RNA Clean & Concentrator-5 kit (Zymo Research, Cat# R1015) to remove enzyme and reaction components. Reverse transcription was performed using SuperScript IV reverse transcriptase (Thermo Fisher Scientific, Cat# 18090010) with a multiplexed pool of gene-specific reverse primers and dNTP mix (New England Biolabs, Cat# N0447S). Target regions were amplified using NEBNext Ultra II Q5 Master Mix (New England Biolabs, Cat# M0544) with pooled forward and reverse primers. Indexed sequencing libraries were generated by a second PCR using universal P5 and P7 indexing primers (Takara Bio, Cat# 634413). Libraries were assessed using an Agilent High Sensitivity D1000 ScreenTape assay and purified with AM-Pure XP beads (Beckman Coulter, Cat# A63881) prior to sequencing on an Illumina platform.

#### Ribosome profiling data analysis

Bovine embryo ribosome dataset was downloaded from GSE196484.^36^ Raw sequencing data were first trimmed using Cutadapt (v5.0) to discard adapter sequences. Trimmed reads were then mapped to bosTau9 (ARS_UCD1.3) using STAR (v2.7.9a). Uniquely mapped reads were assigned to genes using HTseq-count (v2.0.5), and normalization was performed using edgeR package. F6 to F10 were used to calculate the expression levels of each gene in polysome-bound fraction at different time points.

#### m^6^A site editing using CRISPR PEmax

Bovine embryos were produced via *in vitro* fertilization (IVF) and collected at the zygote stage (16–18 h post fertilization) for prime editing. The optimized prime editing system, PEmax, was delivered via cytoplasmic microinjection of *in vitro* produced PEmax mRNA and a synthetic prime editing guide RNA (pegRNA) designed for the target locus. Microinjections were performed in BOWASH (IVF Bioscience) medium using a micromanipulator-equipped inverted microscope. Following microinjection, embryos were cultured in IVF-BIO IVC medium at 38.5°C under a humidified atmosphere of 5% CO_2_, 5% O_2_, and 90% N_2_ until the blastocyst stage. Individual blastocysts were collected for genomic DNA (gDNA) extraction using the Red Extract-N-Amp kit (Sigma XNAT-100RXN) as previously described. Briefly, each embryo was lysed in 4 μL extraction buffer and 1 μL tissue preparation buffer at room temperature for 15 min, followed by heat inactivation at 95°C for 5 min. Samples were cooled to room temperature before adding 4 μL neutralization buffer, and DNA was stored at 4°C for no more than three days before subsequent PCR analysis. Editing efficiency was assessed via Sanger sequencing or next-generation sequencing (NGS), with quantification based on the percentage of successfully edited alleles.

#### Plasmid preparation, *in vitro* transcription, and microinjection

*In vitro* transcribed H2b:GFP RNA was prepared from a plasmid using the T3 mMessage mMachine Kit (Ambion), and it was used as an injection control. Plasmids pMSCV-dCasRx-ALKBH5 H204A (Mutant) and pMSCV-dCasRx-ALKBH5 (Wild-Type) were used as templates for *in vitro* transcription. A T7 overhang forward primer (Data S1) was used in PCR to amplify templates from the above plasmids for transcription. The PCR products were purified using the GeneElute PCR Purification Kit (NA1020, Sigma), followed by *in vitro* transcription and poly(A) tailing using the HiScribe^®^ T7 ARCA mRNA Kit (New England Biolabs, Ipswich, MA). The RPL12 wild-type gene was subcloned into the pGEMHE vector (Addgene #105519) by replacing the TRIM21 sequence to generate the final construct pGEMHE_mEGFP_WTRPL12 (cloning performed by Azenta Life Sciences). This plasmid was linearized with PacI and purified using the GeneElute PCR Purification Kit (NA1020, Sigma) for use in *in vitro* transcription.

Zygote-stage embryos were microinjected with the *in vitro* transcribed H2B:GFP RNA in combination with CRISPR reagents. Microinjections were performed using a FemtoJet 4i microinjector (Eppendorf), an inverted microscope (Leica DMi8), and micromanipulators (Narishige). Embryos collected between 18 and 20 h post-IVF were used for injection. The injection solution contained 20 ng/μL of H2B:GFP RNA along with CRISPR components. Injected presumptive zygotes were cultured in 50 μL BO-IVC medium for further development, and embryo progression was monitored using a Leica DMI 6000 B inverted microscope.

#### Nascent protein detection

To visualize nascent protein synthesis, Click-iT HPG reagents (Invitrogen) were used. Embryos were incubated in 50 μM Click-iT HPG working solution in methionine-free medium for 2 h and processed according to the manufacturer’s protocol. Briefly, embryos were fixed with 3.7% formaldehyde in PBS for 15 min, followed by permeabilization with 0.5% Triton^®^ X-100 for 20 min at room temperature. After three PBS washes, embryos were incubated with a Click-iT reaction cocktail containing Alexa Fluor-conjugated azide for 30 min (protected from light). Embryos were then mounted on slides using ProLong Gold Antifade Mountant with DAPI and scanned using a Leica SP5 confocal microscope. For the negative control, embryos were treated with cycloheximide (50 μg/mL) prior to HPG labeling.

#### RT–qPCR-based m^6^A identification

Site-specific m^6^A levels were quantified following previously published protocols^57,58^ with minor modifications. Total RNA was extracted from edited embryos (at least 10 embryos per replicate per group) using TRIzol (Sigma), with glycogen added to enhance RNA recovery. The RNA was treated with DNase I (Invitrogen) at 37°C for 30 min, followed by purification using the RNeasy MinElute Kit (Qiagen). Two reverse transcription reactions were prepared using either Bst DNA polymerase (NEB) or SuperScript III (Invitrogen), followed by PCR amplification and quantification exactly as described in the referenced protocol.^57^

#### RNA sequencing analysis of m^6^A-RPL edited embryos

Five to ten 4- or 8-cell embryos were pooled for RNA-seq library preparation. Embryos and cells were used directly for library preparation without RNA extraction following SMART-Seq v4 Ultra Low Input RNA kit (Takara, Mountain View, CA) manufacturer’s instructions. Pooled indexed libraries were then sequenced on the Illumina NovaSeq 6000 platform with 150-bp paired-end reads. RNA-seq analysis was conducted as described above.

### QUANTIFICATION AND STATISTICAL ANALYSIS

Statistical differences between pairs of datasets were analyzed by two-tailed unpaired t-tests. Values of *p* < 0.05 were considered statistically significant. Where applicable, all quantitative data were presented as the mean ± standard error or the mean. The number of biological replicates was indicated as ‘n’ in figure legends. Gene ontology enrichment analyses were performed using the goseq R package.^59^ The Benjamini-Hochberg (BH) procedure was used for multiple testing correction.

## Supplementary Material

1

2

Supplemental information can be found online at https://doi.org/10.1016/j.celrep.2026.117635.

## Figures and Tables

**Table:** KEY RESOURCES TABLE

REAGENT or RESOURCE	SOURCE	IDENTIFIER
Antibodies		
No Antibodies used in this manuscript	N/A	N/A
Chemicals, peptides, and recombinant proteins		
BO-IVM medium	IVF Bioscience	Cat. No.#71001
BO-SemenPrep medium	IVF Bioscience	Cat. No.#71003
BO-IVC Medium	IVF Bioscience	Cat. No.#71005
WASH	IVF Bioscience	Cat. No.#51002
BO-IVF	IVF Bioscience	Cat. No.#71004
Oil for embryo culture	Fujifilm	Cat. No.#9305
TRI reagent	Sigma-Aldrich	Cat. No.#T9424
Chloroform	Sigma-Aldrich	Cat. No.#C2432
Isopropanol	Fisher Chemical	Cat. No.#A451SK-4
Ethanol	Fisher Chemical	Cat. No.#BP2818-500
Formaldehyde	Electron Microscopy Sciences	Cat. No.#15710
PBS	Sigma-Aldrich	Cat. No.#D8537-1 L
Triton^®^ X-100	Sigma-Aldrich	Cat. No.#T8787-200ML
ProLong Gold Antifade Mountant with DAPI	Invitrogen	Cat. No.#P36931
Cycloheximide	Thermo Scientific	Cat. No.#J66004.XF
Glycogen	Thermo Scientific	Cat. No.#R0551
DNase I	Invitrogen	Cat. No.#AM2239
BstI polymerase	NEB	Cat. No.#M0328S
SuperScript III	Invitrogen	Cat. No.#18080093
MjDim1	Wang et al.,^12^	Gift from Chuan He lab
Allyl-SAM	Wang et al.,^12^	Gift from Chuan He lab
Tris–HCl	Thermo Scientific	Cat. No.#15568025
SUPERase·In RNase inhibitor	Invitrogen	Cat. No.#AM2696
Nuclease P1	New England BioLabs	Cat. No.#M0660S
Fast AP	Thermo Fisher Scientific	Cat. No.#EF0651
RNase H	New England BioLabs	Cat. No.#M0297L
RNaseOUT	Invitrogen	Cat. No.#10777019
EDTA	Thermo Fisher Scientific	Cat. No.#15575020
T4 Polynucleotide Kinase	New England BioLabs	Cat. No.#M0201L
PEG 8000	New England BioLabs	Cat. No.#M0373L
T4 RNA Ligase 2, truncated KQ	New England BioLabs	Cat. No.#M0373L
5′ deadenylase	New England BioLabs	Cat. No.#M0331S
RecJf	New England BioLabs	Cat. No.#M0264L
Reverse transcriptase, recombinant HIV	Worthington Biochemical	Cat. No.#LS05006
Actinomycin D	Sigma-Aldrich	Cat. No.#A1410
SDS	Invitrogen	Cat. No.#AM9820
DMSO	Sigma-Aldrich	Cat. No.#D2653
Hexaamminecobalt(III) chloride Co(NH3) 6Cl 3	Sigma-Aldrich	Cat. No.#481521
NEBNext Ultra II Q5 Master Mix	New England BioLabs	Cat. No.#M0544L
ZnCl 2	Sigma-Aldrich	Cat. No.#Z0152
MgCl 2	Sigma-Aldrich	Cat. No.#M4880
NH4Cl	Sigma-Aldrich	Cat. No.#1.01142
KI	Sigma-Aldrich	Cat. No.#207969
I 2	Sigma-Aldrich	Cat. No.#207772-5G
Na2S2O3	Sigma-Aldrich	Cat. No.#217263-5G
Recombinant DNA		
pMSCV-dCasRx-ALKBH5 H204A (Mutant)	Wang et al.,^12^	N/A
pMSCV-dCasRx-ALKBH5 (Wild-Type)	Wang et al.,^12^	N/A
pGEMHE vector	Addgene	Cat. No.#105519
pGEMHE_mEGFP_WTRPL12	This Paper	N/A
Critical commercial assays		
NEBNext Ultra II RNA Library Prep Kit	New England Biolabs	Cat. No.#E7770L
Red Extract-N-Amp kit	Sigma-Aldrich	Cat. No.# XNAT-100RXN
T3 mMessage mMachine Kit	Invitrogen	Cat. No.#AM1338
HiScribe^®^ T7 ARCA mRNA Kit	New England Biolabs	Cat. No.#E2060S
GeneElute PCR Purification Kit	Sigma-Aldrich	Cat. No.#NA1020
Click-iT HPG reagents	Invitrogen	Cat. No.#C10338
RNeasy MinElute Kit	Qiagen	Cat. No.#74106
SMART-Seq v4 Ultra Low Input RNA kit	Takara	Cat. No.#740902.1
Deposited data		
SAC-seq	This paper	GSE301371
RNA-seq	This paper	GSE301330
Software and algorithms		
Cutadapt	N/A	v5.0
Bowtie2	N/A	v2.2.8
Samtools	N/A	v1.18
Blastn	N/A	v2.3.0+
STAR	N/A	v2.7.9a
UMI-tools	N/A	v1.1.6
bedtools	N/A	v2.26.0
metaPlotR	github	N/A
Htseq-count	N/A	v2.0.5
Other		
3′ Adaptor	Wang et al.,^12^	Gift from Chuan He lab
cDNA adaptor	Wang et al.,^12^	Gift from Chuan He lab
Spike-in probes and spike-in mix	Wang et al.,^12^	Gift from Chuan He lab

## Highlights

- SAC-seq maps m^6^A dynamics in bovine embryos at single-nucleotide resolution
- Reveals coordinated and uncoupled relationships between m^6^A and gene expression
- Integrates m^6^A, transcription, and translation of ribosomal protein genes
- m^6^A site demethylation in RPL12 impairs zygotic genome activation and embryo development
