# Supplementary material for: Single-nucleotide RNA m6A mapping in bovine preimplantation development reveals site-specific regulation of RPL12 at zygotic genome activation
Source: Cell Rep. Author manuscript; Available in PMC 2026 Aug 23. (PMC13500050; doi:10.1016/j.celrep.2026.117635)

Cell Reports, Volume *45*

Supplemental information

Single-nucleotide RNA m^{6}A mapping in bovine preimplantation development reveals site-specific regulation of RPL12 at zygotic genome activation

Rajan Iyyappan, Yichi Niu, Yang Li, Hao Ming, Kinga Pajdzik, Noah R. Rakestraw, Piyush K. Jain, Chuan He, Chenghang Zong, and Zongliang Jiang

Supplementary Figure Legends

Supplementary Figure 1. Validation of m^6A sites and assessment of motif distribution across sequencing depth thresholds. **A**. m^6A levels measured by low input eTAM-seq for selected transcripts at GV and blastocyst stages. **B**. m^6A levels measured by SAC-seq for selected transcripts at GV and blastocyst stages. **C-D**. Fraction of m^6A sites within DRACH and RRACH motifs across developmental stages under increasing read depth thresholds.

Supplementary Figure 2. Subclustering analysis of m^6A-marked transcripts in comparison with total transcript abundance and translatome profiles across clusters 1–6. A–F. Heatmaps showing stage-specific patterns of m^6A dynamics, transcript abundance, and ribosome occupancy for each subcluster within cluster 1 (**A**), cluster 2 (**B**), cluster 3 (**C**), cluster 4 (**D**), cluster 5 (**E**), and cluster 6 (**F**). Subclusters are labeled Sub1–Sub5. Developmental stages are shown from GV to BL.

Supplementary Figure 3. Expression-normalized m^6A site density across subclusters. **A**. Boxplots showing the distribution of expression-normalized m^6A site density across subclusters within each cluster. Statistical significance between subclusters is indicated. **B**. Distribution of m^6A site density (number of m^6A sites per kb per RPKM) across subclusters (Sub1–Sub5) within each cluster (Clusters 1–6) across developmental stages (GV to BL).

Supplementary Figure 4. Motif specific m^6A dynamics in non-coding RNAs. **A**. Heatmaps showing Z-score normalized m^6A levels of lncRNAs stratified by DRACH and non-DRACH motifs across developmental stages (GV to blastocyst). **B**. Heatmaps showing Z-score normalized m^6A levels of snoRNAs stratified by DRACH and non-DRACH motifs across developmental stages.

Supplementary Figure 5. Validation of m^6A modification at the RPL12 A148 site. Relative m^6A levels at the RPL12 A148 site measured by eTAM-seq in ALKBH5 wild-type (WT) and ALKBH5 mutant (Mut) samples.

Supplementary Figure 6. RNA-seq analysis on control and PEMax-edited embryos at the 4-cell and 8-cell stages. **A**. Volcano plots showing differentially expressed total transcripts at 4-cell stage. **B**. Volcano plots showing differentially expressed total transcripts at 8-cell stage. **C**. Volcano plots showing differentially expressed total transcripts between 4-cell and 8-cell stages in control. **D**. Volcano plots showing differentially expressed total transcripts between 4-cell and 8-celll stage at edited RPL12 A148G. **E-F**. Volcano plots showing differentially expressed ribosomal protein genes (E) and translation initiation/elongation factor (F) between 4-cell and 8-cell stages in control embryos. **G-H**. Volcano plots showing differentially expressed ribosomal protein genes (G) and translation initiation/elongation factors (H) between 4-cell and 8-cell stages in RPL12 A148G–edited embryos.

Supplementary Figure 1

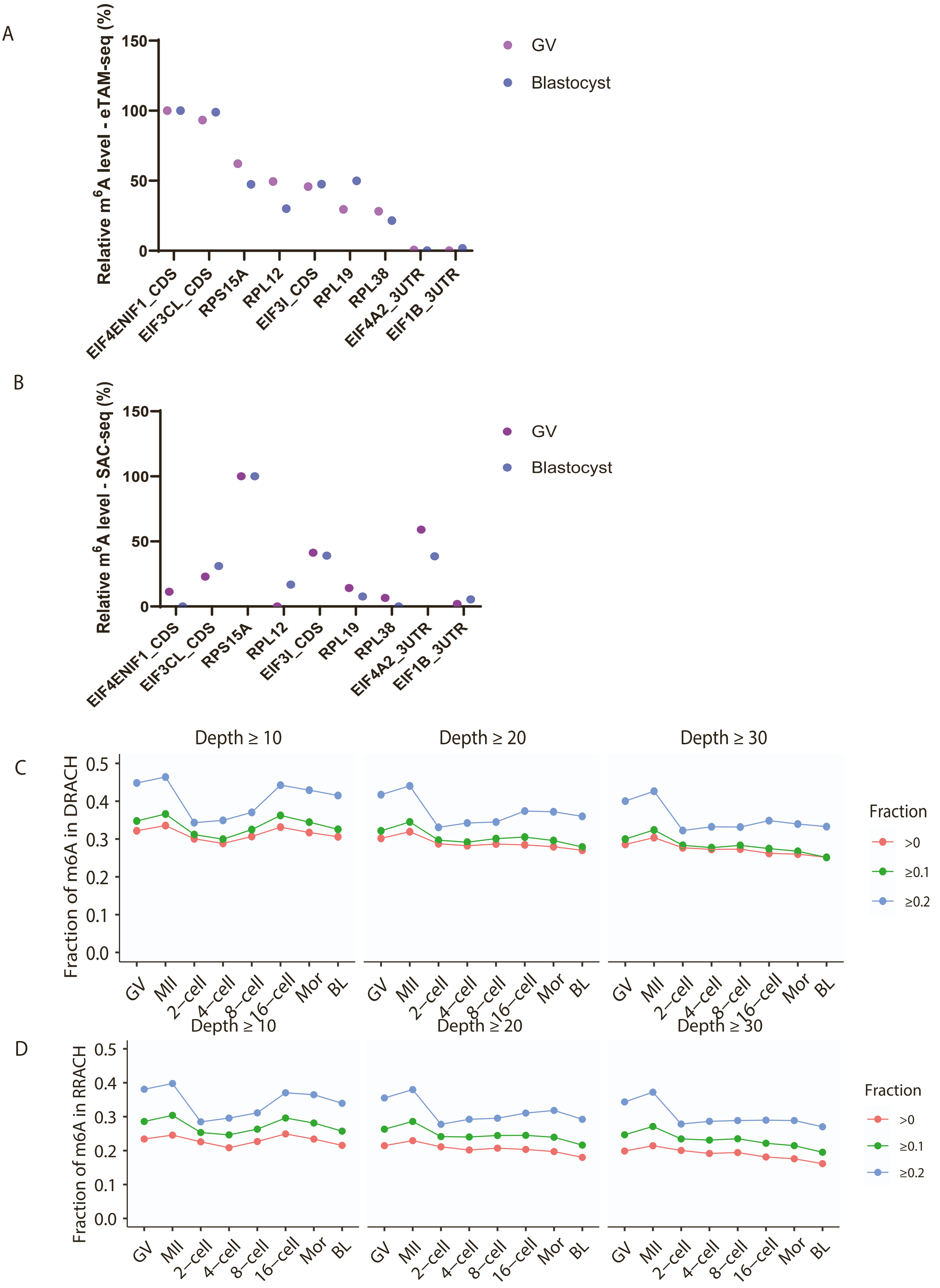

Supplementary Figure 2

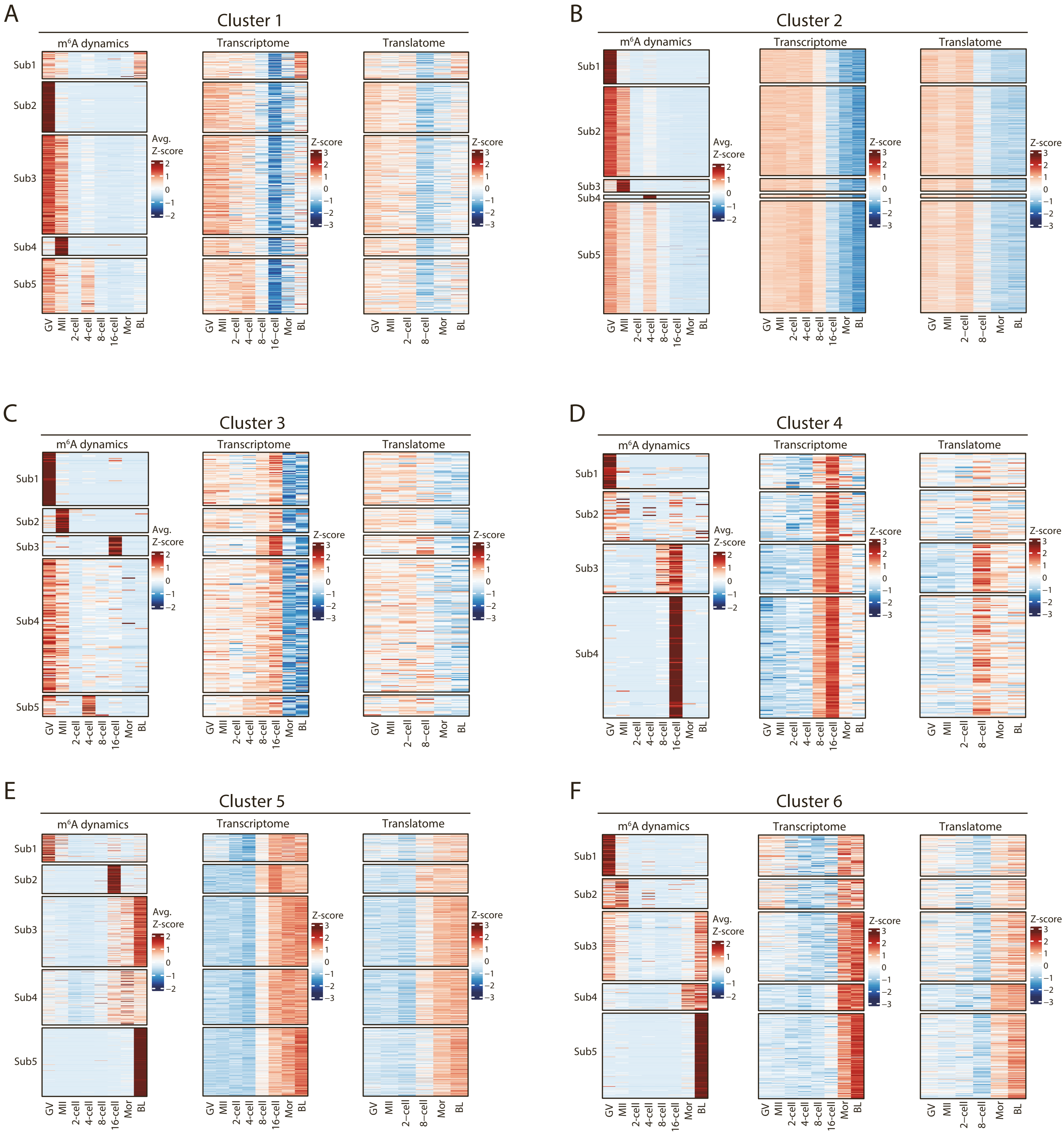

Supplementary Figure 3

A

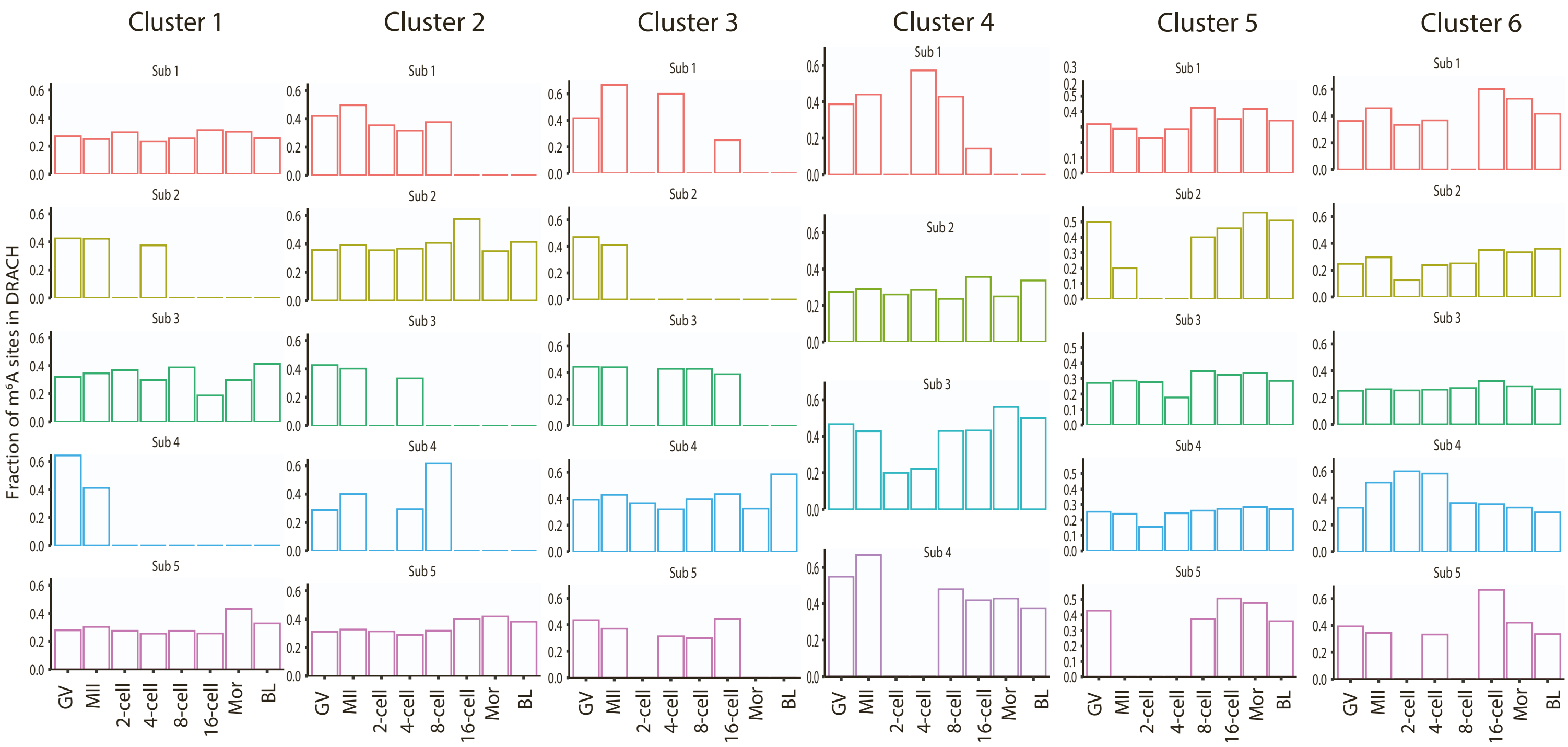

B

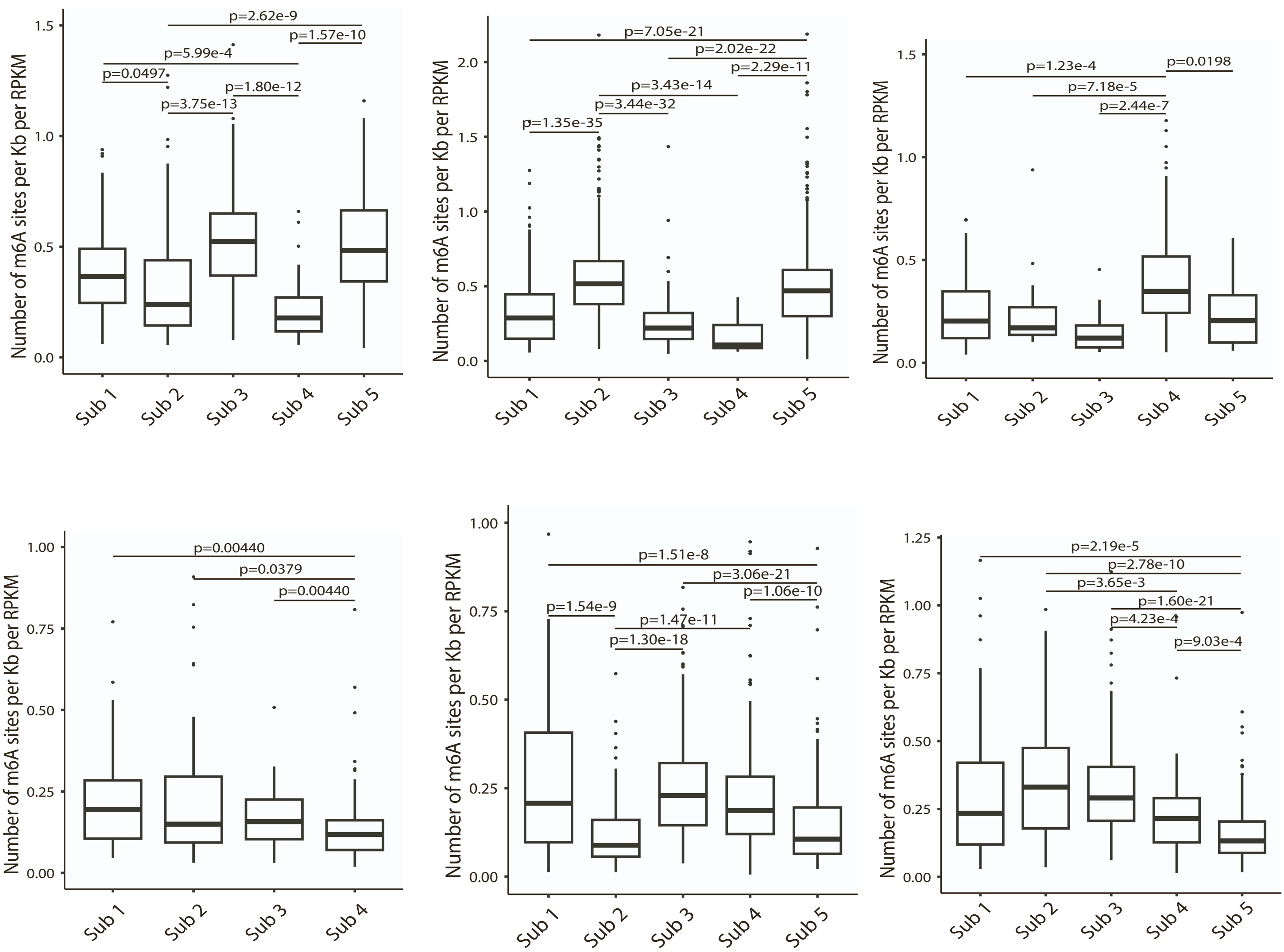

Supplementary Figure 4

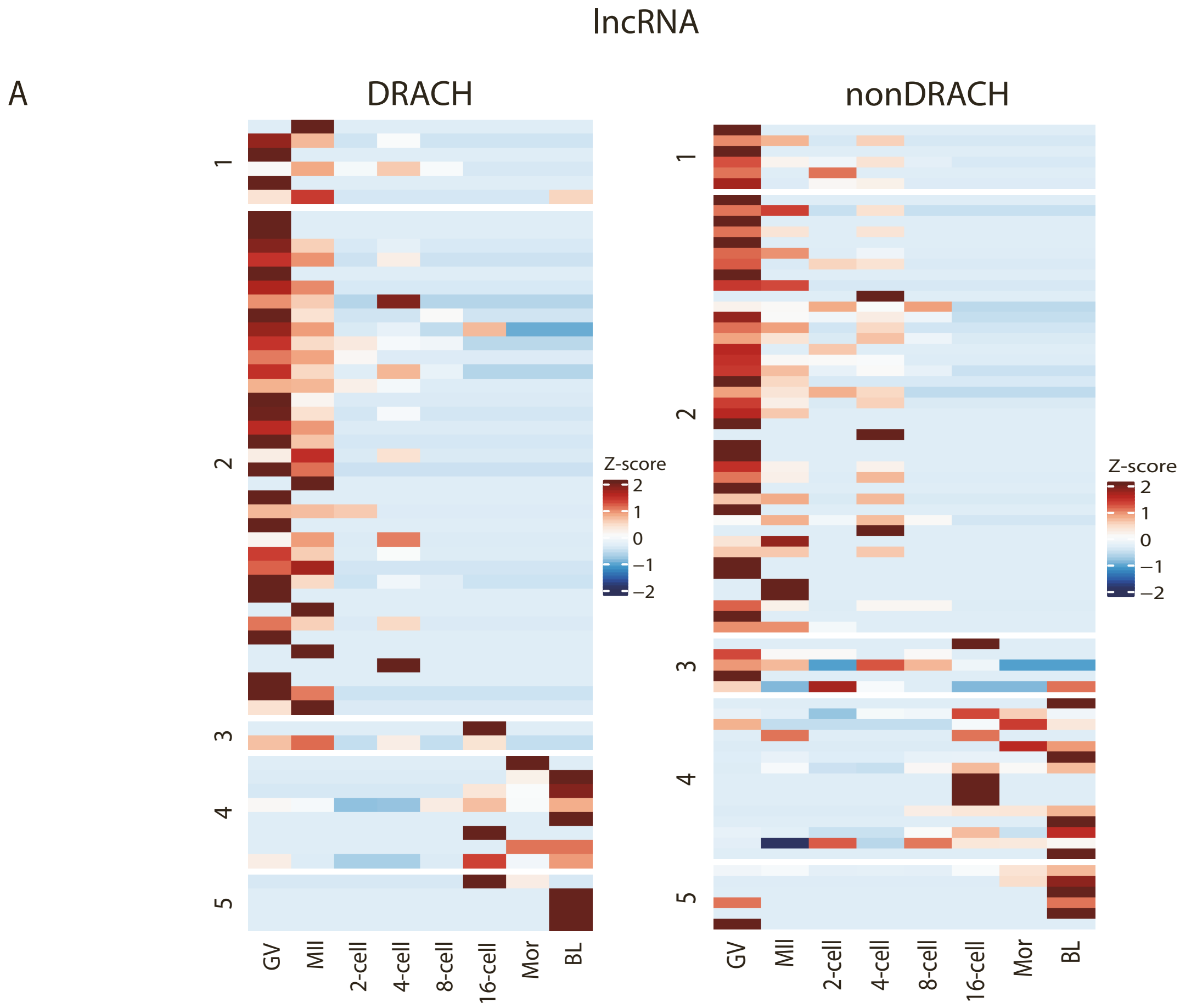

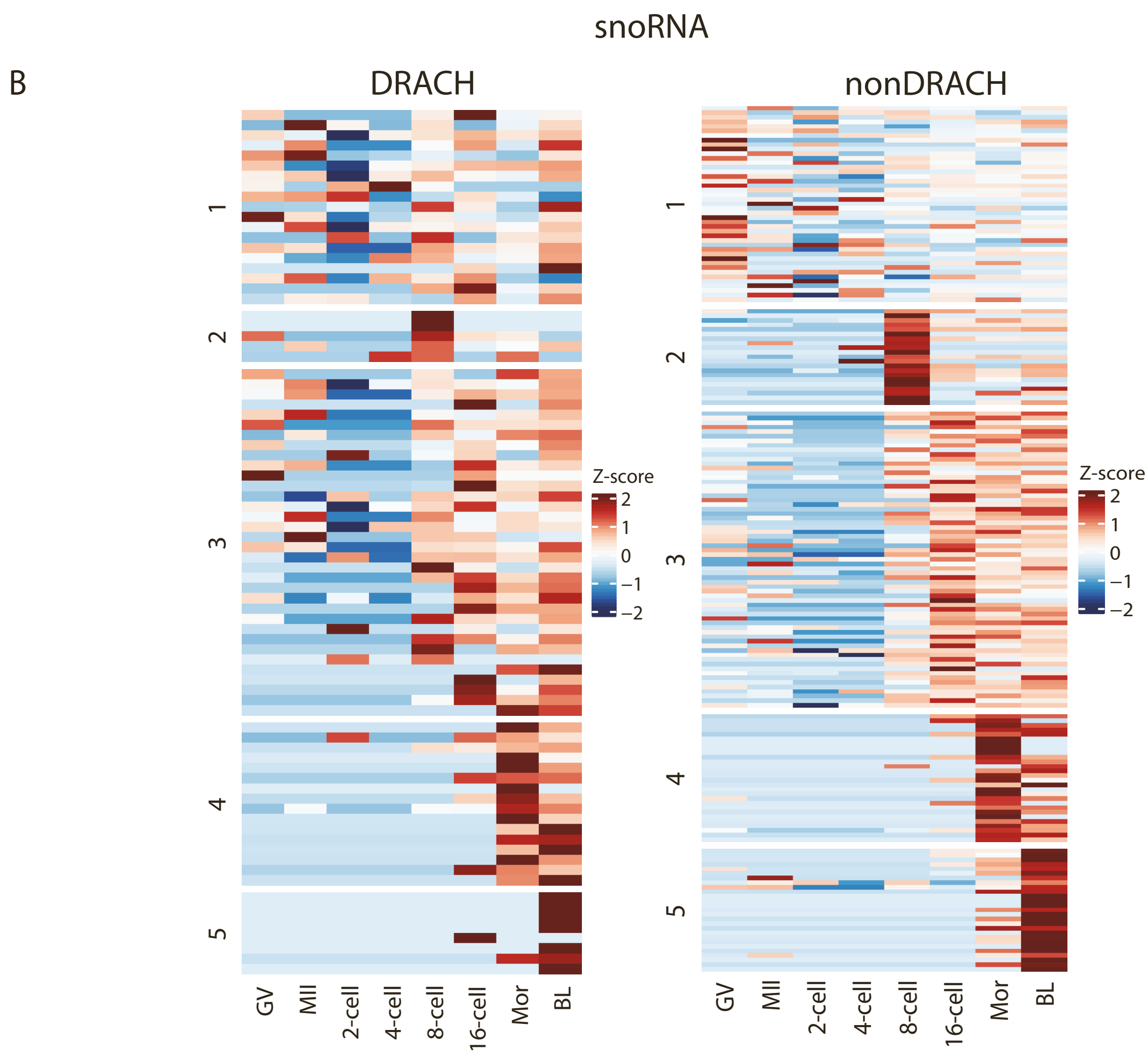

Supplementary Figure 5

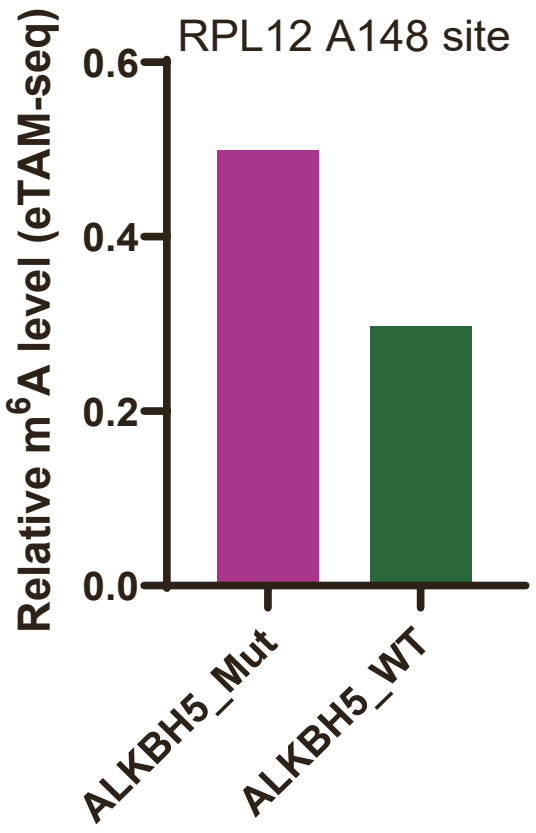

Supplementary Figure 6

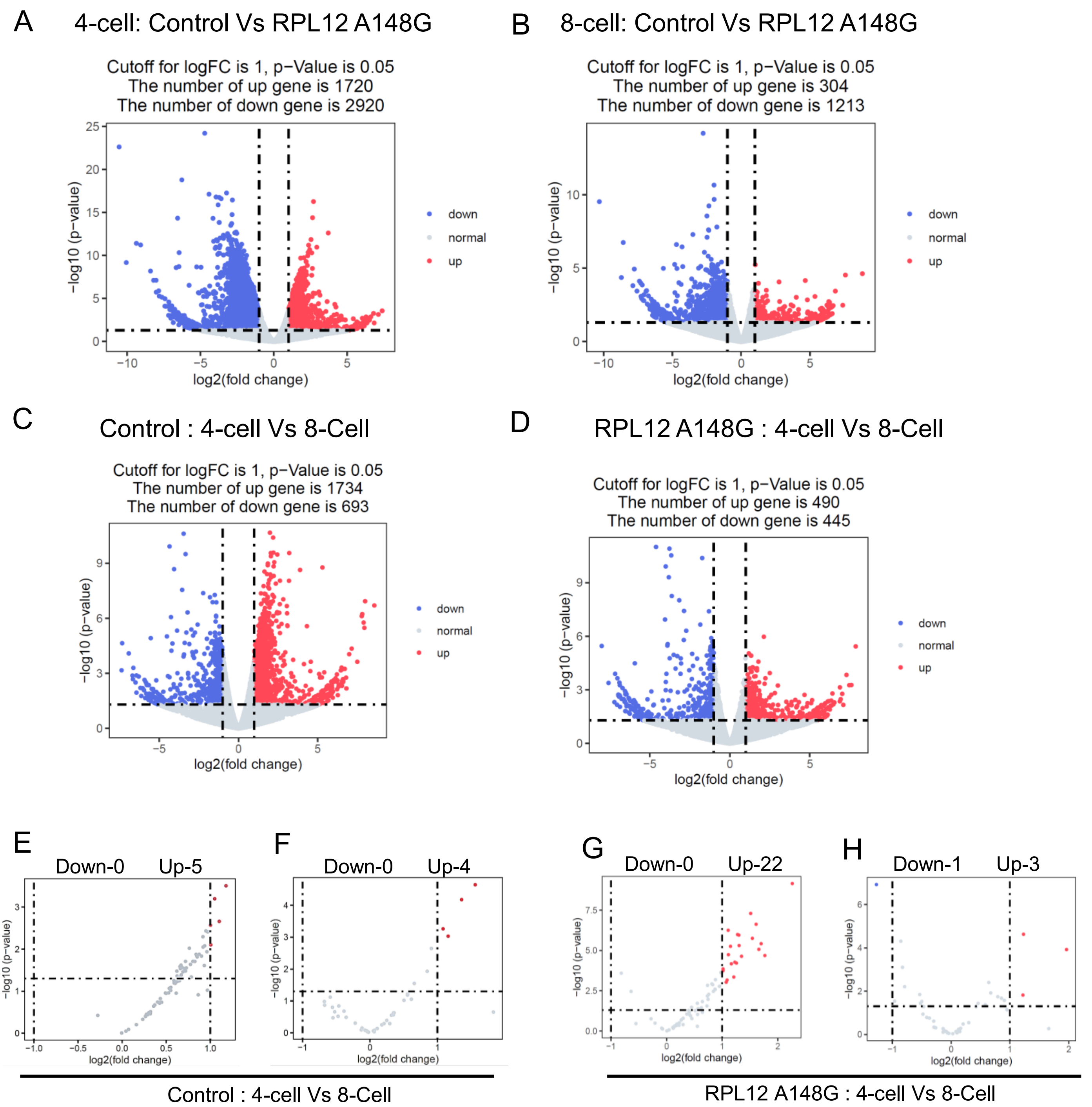

Supplement: Supplementary file 1 [file NIHMS2199957-supplement-1.pdf]
